# Single‐cell transcriptome analysis deciphers the CD74‐mediated immune evasion and tumour growth in lung squamous cell carcinoma with chronic obstructive pulmonary disease

**DOI:** 10.1002/ctm2.1786

**Published:** 2024-08-07

**Authors:** Denian Wang, Sixiang Li, Zhi Yang, Chunyan Yu, Pengfei Wu, Ying Yang, Rui Zhang, Qingyan Li, Jian Yang, Hongchun Li, Guiyi Ji, Yan Wang, Kang Xie, Yanyan Liu, Kaige Wang, Daxing Zhu, Wengeng Zhang, Dan Liu, Bojiang Chen, Weimin Li

**Affiliations:** ^1^ Precision Medicine Research Center Precision Medicine Key Laboratory of Sichuan Province State Key Laboratory of Respiratory Health and Multimorbidity West China Hospital Sichuan University Chengdu Sichuan China; ^2^ Department of Respiratory and Critical Care Medicine Precision Medicine Center Frontiers Science Center for Disease‐Related Molecular Network West China Hospital Sichuan University Chengdu Sichuan China; ^3^ Research Units of West China Chinese Academy of Medical Sciences West China Hospital Chengdu Sichuan China; ^4^ Department of Respiratory and Critical Care Medicine National Clinical Research Center for Respiratory Disease The First Affiliated Hospital of Guangzhou Medical University Guangzhou Guangdong China; ^5^ Department of Nephrology West China Hospital Sichuan University Chengdu Sichuan China; ^6^ Frontiers Science Center for Disease‐Related Molecular Network Laboratory of Omics Technology and Bioinformatics West China Hospital Sichuan University Chengdu Sichuan China; ^7^ Department of Respiratory Health Frontiers Science Center for Disease‐Related Molecular Network West China Hospital Sichuan University Chengdu Sichuan China; ^8^ Center of Growth Metabolism, and Aging Key Laboratory of Bio‐Resources and Eco‐Environment College of Life Sciences Sichuan University Chengdu Sichuan China; ^9^ National Chengdu Center for Safety Evaluation of Drugs State Key Laboratory of Biotherapy/Collaborative Innovation Center for Biotherapy West China Hospital Sichuan University Chengdu Sichuan China; ^10^ Health Management Center West China Hospital Sichuan University Chengdu Sichuan China; ^11^ Department of Thoracic Surgery West China Hospital Sichuan University Chengdu Sichuan China; ^12^ Lung Cancer Center West China Hospital Sichuan University Chengdu Sichuan China

**Keywords:** CD74, chronic obstructive pulmonary disease, immune evasion, lung squamous cell carcinoma, single‐cell RNA sequencing

## Abstract

**Background:**

Chronic obstructive pulmonary disease (COPD) contributes to the incidence and prognosis of lung cancer. The presence of COPD significantly increases the risk of lung squamous cell carcinoma (LSCC). COPD may promote an immunosuppressive microenvironment in LSCC by regulating the expression of immune‐inhibitory factors in T cells, although the mechanisms remain unclear. In this study, we aimed to decipher the tumour microenvironment signature for LSCC with COPD at a single‐cell level.

**Methods:**

We performed single‐cell RNA sequencing on tumour tissues from LSCC with or without COPD, then investigated the features of the immune and tumour cells. We employed multiple techniques, including multispectral imaging, flow cytometry, tissue microarray analysis, survival analysis, co‐culture systems and in vitro and in vivo treatment experiments, to validate the findings obtained from single‐cell analyses.

**Results:**

LSCC with COPD showed increased proportions of tumour‐associated macrophages (TAMs) and higher levels of CD8^+^ T cell exhaustion molecules, which contributed to an immunosuppressive microenvironment. Further analysis revealed a critical cluster of CD74^+^ tumour cells that expressed both epithelial and immune cell signatures, exhibited a stronger capacity for tumorigenesis and predicted worse overall survival. Notably, migration inhibitory factor (MIF) secreted by TAMs from LSCC with COPD may promote the activation of CD74. MIF‐CD74 may interact with CD8^+^ T cells and impair their anti‐tumour activity by regulating the PI3K‐STAT3‐programmed cell death‐1 ligand 1 signalling pathway, facilitating tumour proliferation and immune evasion.

**Conclusions:**

Our comprehensive picture of the tumour ecosystem in LSCC with COPD provides deeper insights into relevant immune evasion mechanisms and potential targets for immunotherapy.

**Highlight:**

- Our results demonstrated higher proportions of tumour‐associated macrophages (TAMs) and higher levels of exhaustion molecules in CD8^+^ T cells in the microenvironment of LSCC with COPD.
- CD74^+^tumour cells were associated with poor disease prognosis.
- Migration inhibitory factor (MIF)‐CD74 may interact with CD8^+^ T cells and impair their anti‐tumour activity by regulating the PI3K‐STAT3‐PD‐L1 signalling pathway, facilitating immune evasion.

## Graphical abstract

- A higher prevalence of tumour‐associated macrophages (TAMs) alongside elevated levels of exhaustion markers in CD8^+^ T cells, which together promote an immunosuppressive milieu in LSCC with COPD.
- A critical cluster of CD74^+^ tumour cells was associated with poor disease prognosis.
- MIF may activate CD74 on tumour cells from LSCC with COPD, enhancing their malignant potential.
- The interaction between MIF‐CD74 and CD8^+^ T cells appears to suppress these T cells' anticancer activity by modulating the PI3K‐STAT3‐PD‐L1 signalling pathway, thereby aiding tumour evasion of immune responses.

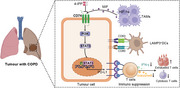

## 1 INTRODUCTION

Lung cancer (LC) continues to be the principal cause of cancer‐related mortality globally, marked by a 5‐year survival rate below 20%.^1^, ^2^ Although diagnostic and therapeutic advancements have been made, patient outcomes have not significantly improved.^3^ The primary subtypes of LC include lung adenocarcinoma and lung squamous cell carcinoma (LSCC), with LSCC comprising about 30% of non‐small cell LC cases.^4^ Studies have shown that about 91% of LSCC occurrences in Asian populations are closely related to smoking history.^5^ For LC‐targeted therapies, adenocarcinoma shows more significant benefits, whereas the results for smoking‐related LSCC cases have not been promising.^5^ Therefore, the pathogenesis of LSCC may be more complex and heterogeneous.

Smoking is also a major risk factor for chronic obstructive pulmonary disease (COPD).^6^ COPD is a chronic inflammatory disease of the airways characterized by immune‐mediated airway remodelling and destruction of lung parenchyma.^1^ The abnormal immune microenvironment resulting from COPD not only leads to excessive oxidative stress and harmful lung remodelling but also significantly increases the susceptibility and progression of LSCC.^7^, ^8^, ^9^ Studies have shown that some LSCC patients who smoke may significantly benefit from immunotherapy.^10^ Hence, immune evasion plays a crucial role in LSCC pathogenesis.^11^ Because of this, a better understanding of the biological mechanisms underlying LC cases that coexist with COPD is necessary.^12^

Recent advancements have been made with elucidating the immune microenvironment in LSCC tumours that coexist with COPD.^10^, ^13^ Previous studies have shown that T cells in LSCC coexisting with COPD significantly express the immunosuppressive receptor programmed cell death‐1 (PD‐1).^10^, ^13^ Increased PD‐1 expression levels can result in T cell exhaustion but also can lead to an enhanced response to immunotherapies that target PD‐1/PD‐1 ligand 1 (PD‐L1) signalling.^14^, ^15^ However, little is known about how COPD impacts the heterogeneity and tumour microenvironment (TME) of LSCC at a single‐cell level.^16^, ^17^

To better elucidate the complex interactions between COPD and LSCC, we used single‐cell RNA sequencing (scRNA‐seq) to analyse the transcriptome profiles of immune cells and tumour cells in LSCC with COPD. We observed higher proportions of TAMs, upregulated expression levels of MIF by HIF‐1α and high levels of exhausted molecules in CD8^+^ T cells. These factors collectively contribute to an immunosuppressive microenvironment. Remarkably, we identified a critical cluster of CD74^+^ tumour cells that exhibited both epithelial and immune signatures in LSCC with COPD. This cluster of cells was also associated with poor disease prognosis. Notably, our study demonstrated that MIF secreted by TAMs from LSCC with COPD may promote the activation of CD74. MIF‐CD74 significantly increased phosphorylation of molecules in the PI3K/STAT3 pathway, resulting in upregulated PD‐L1 expression levels and suppressed CD8^+^ T cell function. Therefore, our research provides a detailed analysis of the multicellular ecosystem in LSCC with COPD, highlighting a significant population of CD74^+^ tumour cells. This discovery suggests that targeting CD74^+^ cells may be an effective strategy for treating LSCC.

## 2 MATERIALS AND METHODS

### 2.1 Collection of lung cancer tissues

Our LC tissue samples were obtained from 14 patients at the Thoracic Surgery and Lung Cancer Center of West China Hospital of Sichuan University. COPD was diagnosed and its severity assessed using Global Initiative for Chronic Obstructive Lung Disease criteria.^18^ Paired fresh tumour samples (approximately 2 cm × 2 cm × 1 cm) and adjacent normal tissue samples (at least 2 cm from the tumour) were collected from each patient. These samples were obtained from patients with untreated primary LSCC.

Among them, 16 samples from 8 patients were collected between February 2018 and February 2019 (refer to Table 1 and Table S1 for details). Each sample was collected immediately after surgical excision and divided into three parts: one for preparing a single‐cell suspension, one for fluorescence‐activated cell sorting (FACS) analysis and one for immunohistochemistry (IHC) and multiplex immunofluorescence (mIF) staining.

**TABLE 1:** Baseline characteristics of the patients with lung squamous cell carcinoma (LSCC).

Characteristics	Tumour	CTumour	*p* Value
Subjects, *n* (%)	7 (50)	7 (50)	
Sex, (*n*)	Male (7)	Male (7)	
Age, year, mean ± SD	58 ± 6	63 ± 6	.161
Smokers, n (%)	7 (100)	7 (100)	
Smoking history, pack‐years, mean ± SD	6 ± 7.3	25 ± 11	.002
Histologic subtype (*n*)	LUSC (7)	LUSC (7)	
pTNM stages, *n* (%)			
I‐II	2 (29)	3 (43)	.577
III‐IV	5 (71)	4 (57)	
Pneumonectomy, *n* (%)	7 (100)	7 (100)	
% FEV_1_/FVC, mean ± SD	81 ± 2	47 ± 5	<.0001
FEV_1_ (% of a predicted value), mean ± SD	112 ± 13	56 ± 7	<.0001

*Note*: All variables were evaluated among the 14 patients with LSCC. Quantitative data between Tumour and CTumour groups were analysed by Student’ *t* test. Categorical data were analysed by chi‐square test.

Abbreviations: COPD, chronic obstructive pulmonary disease; CTumour, tumour with COPD; FVC, forced vital capacity; LUSC, lung squamous cell carcinoma; M, metastasis. FEV1, forced expiratory volume at the first second; N, lymph node; T, tumour; Tumour, Tumour without COPD.

Between January 2020 and December 2020, we collected samples from six patients immediately following surgical excision. Each sample was bifurcated for distinct analyses; one part was used for FACS and the other for IHC and mIF staining. Comprehensive clinical and pathological details are provided in Table 1 and Table S1. The express written consent of all study subjects was obtained. This study followed the ethical guidelines of West China Hospital of Sichuan University and was officially supported by its ethics committee to ensure the ethical compliance of the research process.

### 2.2 Tissue microarrays

LUSC (Cat HLugS180Su08) was purchased from SHANGHAI OUTDO BIOTECH in China. For the human tissue microarrays, the CD74 expression score was calculated as follows: [percentage of CD74‐positive cancer cells in an entire core section (ranging from 0 to 100) multiplied by staining intensity (ranging from 0 to 4)].^18^

### 2.3 Preparation of single‐cell suspensions

In the operating room, it is rapidly converted into a suspension of single cells in just 2–3 h after surgery. MACS tumour dissociation kit (product number 130‐095‐929) produced by Miltenyi Biotec was used in the preparation of single‐cell suspensions. In summary, the tissue was cut into 2–4 mm pieces, which were then transferred to a MACS tube (product number 130‐096‐334, manufactured by Miltenyi Biotec) containing an enzyme mixture prepared from both H and R enzymes in RPMI‐1640 medium. The digestion procedure was performed using the MACS Dissociator supplied by Miltenyi Biotec Company (Cat 130‐093‐235). Bovine serum albumin .4%, product number C102301, provided by Sangon Biotech. Thereafter, the suspension was subjected to a centrifugal force of 500 *g* for 5 min, and the resulting clear liquid was discarded. To remove red blood cells, an erythrocyte disintegrant (10‐fold concentration) (manufactured by BD Biosciences, product number 555899) was applied according to the manufacturer's guidelines. The death cell removal kit produced by Miltenyi Biotec is used to ensure that the survival rate of cells is maintained at more than 90%.

### 2.4 Single‐cell RNA amplification and library preparation

The 3′ single‐cell RNA sequences of cell suspensions were analysed utilizing a single‐cell A‐chip system, a single‐cell 3′ library, a gel bead kit V2 and an i7 composite kit from 10× Genomics. Each channel is filled with approximately 10 000 cells. The emulsion was deconstructed with reagents, and the extracted complementary DNA (cDNA) was subsequently removed with DYNA‐Beads (10× Genomics, product code 2000048). cDNA) was generated through the process of polymerase chain reaction, and the appropriate number of iterations was determined according to the recovery rate of cells. After the replication of DNA sequence, cDNA molecules undergo cleavage treatment, terminal modification, addition of adenine nucleotide tail, binding with specific adapters and finally form a rich molecular library through the amplification process. A library of 450 BP fragments was normalized, pooled and sequenced using 150 cycles of the NextSeq 500 High Yield Kit V2 run on a NextSeq 500 device at an average final concentration of approximately 30 nM. Illumina is located in San Diego, California and is numbered 5.

### 2.5 Single‐cell RNA data processing

In our study, we made use of the Cell Ranger toolkit (version 3.0), provided by 10× Genomics, for the purpose of deciphering cellular barcodes. Subsequently, the unique molecular identifier (UMI) count matrix (version 2.3.4) was analysed using the R package Seurat (version 2.3.4).^19^ Additionally, to ensure data quality, we applied three assessment methods to the raw gene‐cell‐barcode matrix. We excluded low‐quality cells based on the following criteria: cells with fewer than 500 UMIs, cells with more than 6000 or fewer than 200 genes and cells with more than 10% of UMIs derived from the mitochondrial genome.^20^ Additionally, to minimize errors caused by batch effects, we applied a filtration method to remove genes expressed in low‐quality cells. We used the ‘FindIntegrationAnchors’ function in Seurat to correct for batch effects. For a detailed explanation of our methodologies, please refer to our published articles.^21^

### 2.6 Identification of major cell types and subtypes

In our analysis, we applied principal component analysis (PCA) to categorize the genes based on variability in expression, followed by the UMAP algorithm (executed through the ‘RunUMAP’ function) to synthesize and refine these principal components. During cell clustering, we utilized the ‘FindClusters’ function from the Seurat package, adhering to default parameters. Moreover, we identified high‐variability genes using the ‘FindVariableFeatures’ function, aiding in the determination of primary cell types. To quantify changes in gene expression, we employed the ‘FindMarkers’ function in Seurat, selecting genes that exhibited at least 1.5 times the average expression level.^21^ For cluster annotation, we used at least two established marker genes. Clusters characterized by sparse or diverse marker expression were excluded from further analysis, ensuring the rigour and validity of our findings.

The cell types identified and annotated in our study include epithelial cells (marked by KRT5, KRT15, KRT17, KRT18, WFDC2, CAPS and EPCAM), T cells (CD3D, CD3E, CD3G and CD2), myeloid cells (LYZ, CD68, S100A8, S100A9, CD1E and LAMP3), B cells (CD79A, CD79B, CD19 and MS4A1), fibroblast cells (COL1A1, COL3A1 and FN1), mast cells (GATA2, TPSAB1 and TPSB2) and cycling cells (MKI67 and TYMS). This comprehensive profiling enhances our understanding of the cellular architecture within the TME.

### 2.7 CNV analysis

In our study, we aimed to identify tumour cells within epithelial cell populations using the inferCNV method. By comparing the copy number variation (CNV) scores of epithelial cells in tumour samples with those from non‐tumour‐derived epithelial cells, we were able to pinpoint subsets of malignant tumour cells. This approach provided a clear distinction between healthy and cancerous cells, facilitating a deeper understanding of tumour cell characteristics and distribution.

This method identifies regions of the genome with significant differences in copy number variations between the two cell populations. By comparing the inferred tumour cells with the reference cells, inferCNV calculates a CNV score that reflects the likelihood of copy number variations in the tumour‐derived epithelial cells relative to the non‐tumour‐derived epithelial cells.

### 2.8 Gene set variation analysis (GSVA) and ITH score analysis

We primarily conducted pathway analyses using the gene set variation analysis package to assess the pathway activity of individual cells, as previously described.^22^ We defined a significant difference as a Benjamini–Hochberg‐corrected *p*‐value of less than .01.

The ITH score was calculated by averaging Euclidean distance between cells, based on the first 20 components of highly variable genes with normalized expression levels.^23^ The highly variable genes were identified using the FindVariableFeatures function in Seurat.

### 2.9 Inference of tumour cell state by using trajectory analysis

First, we analysed the epithelial cell clusters using scRNA‐seq data from both normal and malignant samples. Malignant cells were selected on the basis of CNV inference. Next, we identified variable genes using the Seurat method.^24^ Subsequently, we conducted trajectory analysis for malignant and normal epithelial cells utilizing the Monocle 2 algorithm.

### 2.10 Expression features of tumour cells

We investigated tumour cells using a nonnegative factorization algorithm, specifically the NMF package.^25^ The NMF algorithm was applied to the relative expression matrix obtained from 10× Genomics, and genes with a standard expression deviation of less than .5 were filtered out. From each cell, we selected 5 primary expression patterns, resulting in a total of 50 expression patterns across the 8 tumours. Then, we utilized the cell scores of each pattern to characterize and identify common patterns among the 50 signatures. These common patterns were further grouped into meta programmes based on the Pearson correlation coefficients calculated between them.

### 2.11 Immune‐related scores for tumour cells

First, we performed the immune meta‐programme analysis using canonical genes selected from Table S10. We calculated the average expressions of immune‐related genes in tumour cells and ranked these genes based on their correlations with the average cell scores.^26^, ^27^ Furthermore, the epithelial scores were calculated on the basis of the expression levels of the following genes: KRT5, KRT6A, KRT7, KRT8, KRT14, KRT15, KRT16, KRT17, KRT18, KRT19, MUC1, SCGB3A2, SFTPB, WFDC2 and EPCAM.

### 2.12 SCENIC analysis

As previously mentioned, single‐cell gene regulatory network analysis and its grouping analysis (referred to as Scenic) are performed.^28^ We have adopted the travel package scheme (version 1). The functional activity of transcription factors (TFs) was evaluated by pooling the RCISTARGET and GRNBOOST motif resources. Gene sequence data from the RCISTARGET toolkit were used to identify the regulatory elements to which the selected TFs specifically bind. The activity of each group of modulators was assessed in each cell using the AUCELL software toolkit. In addition, the combined specificity index of all regulators was estimated by applying the SCFunctions toolkit.

### 2.13 Analysis of intercellular interactions among different cell types

We investigated cell–cell interactions between tumour cells and immune cells in the TME by examining the expressions of a receptor on one cell and its corresponding ligand on another cell, as described in a previous study.^29^ The set of receptor–ligand pairs was obtained from a previous study.^28^ The criteria for determining the ‘expression’ of a ligand or receptor was defined as having an average expression value greater than .2 in a specific cell type. Subsequently, we employed CellPhoneDB to analyse the interactions among different cell types. It is important to note that the cellular network was constructed on the basis of these interactions.

### 2.14 Isolation and culture of TAMs

TAMs were isolated from CTumour and tumour samples using FACS. The cells were stained with antibodies for CD45 (CD45‐PE, BD Pharmingen, Cat 555483), CD68 (CD68‐PE‐Cy7, BD Pharmingen, Cat 565595) and CD11b (CD11b‐APC, BD Pharmingen, Cat 550019) to identify the TAMs.

Subsequently, isolated TAMs were cultured in rpm −1640 medium (Gibco, product code 12633012) containing 10% foetal bovine serum, 1% penicillin and 1% streptomycin for 2–3 days. After treatment with GipZ lentivirus‐mediated human HIF‐1α shRNA or GipZ lentivirus‐mediated human JunB shRNA, TAMs at a concentration of 2 × 10^5^ were inoculated in six‐well plates. Subsequently, the cells were transferred to 96‐well plates with 5 × 10^4^ per well and incubated for 24 h. After this, the cells were reactivated with 20 mg/mL lipopolysaccharide and 20 ng/mL interleukin (IL)‐4 for another 24 h. In the final stage, dilutions were withdrawn and chemokines were quantified using an enzyme‐linked immunosorbent assay (ELISA) kit (manufacturer: R&D, product number: DMF00B) according to the manufacturer's guidelines.

### 2.15 Enzyme‐linked immunosorbent assay (ELISA)

NCI‐H520, KLN205 and THP‐1 were seeded onto 6‐well at a density of 5 × 10^5^, 5 × 10^5^ and 10 × 10^5^ cells, respectively. Then, these cells were exposed to normal oxygen conditions and subjected to oxygen deprivation for 24 and 48 h. Afterwards, we collected the cell supernatant and MIF levels were measured using ELISA kits (R&D, Cat DY91Y1978) according to manufacturer recommendations.

### 2.16 Isolation and culture of mice CD8^+^ T cells

Spleens from OT1 mice were squeezed, and the cell suspension was screened through a 70 µm sieve. Subsequently, the suspension was purified with the aid of a CD8^+^ T cell purification kit (product code CAT) produced by Miltenyi Biotec.130‐096‐543.Refined CD8^+^ T cells were enriched with 2 µg/mL CD3/CD28 (from IBA‐Life Sciences, product number 6‐8920‐050), 10 ng/mL IL‐2 (from R&D, Product No.202‐050) and 10 ng/mL IL‐12 (from PreProTech, Product No.210‐12‐50 UG) were incubated for 48–72 h in RPMI‐1640 medium (purchased from Gibco, Product No.12633012.

### 2.17 CD8^+^ T cells‐mediated tumour cell killing assay

KLN205‐OVA cells (5×10^4^ cells/well) were co‐cultured with activated CD8^+^ T cells (2 × 10^5^ cells/well) at effector‐to‐target ratios of 2, 5 and 10 in 24‐well plates. After incubation, CD8^+^ T cells were collected and centrifuged at 400 *g* for 5 min for the LDH release assay (Abcam). Additionally, KLN205‐OVA cells were collected and counted using trypan blue staining.

### 2.18 Tumour growth analysis and treatment experiment

A total of 2 × 10^6^ KLN205 cells were injected subcutaneously in 150 µL PBS solution in groups of five male nude mice and C57BL/6 mice. The dimensions of the tumour were recorded every 3 days using a vernier caliper. Tumour volume (V) was calculated as (length (mm) × width (mm) height (mm))/2. Tumour tissues were collected on day 15/20.

In the course of the experimental treatment of tumour‐bearing mice, the mice were intraperitoneally administered with a monoclonal antibody against PD‐1 (trade name: BioXcell, product number: BE0146 and antibody clone type: RMP1‐14) or an immunoglobulin prepared from rats (rat Ig, origin: BioXcell and Antibody clonotype: RTK2758). These drugs were injected on days 8, 10, 12 and 14 of the experiment at a dose of 200 µg per mouse diluted in PBS solution.^30^

In the experimental project of synergistic drug administration, C57BL/6 mice were purchased and injected subcutaneously with KLN205 cancer cells at a dose of 2 × 10^6^. After randomization of small experimental animals, an anti‐PD‐L1 immunizing agent was injected intraperitoneally at a dose of 200 µg per animal on days 8, 10, 12 and 14. The small organic molecule 4‐IPP (MedChem Express, product number HY‐110063) was used to block the activity of the MIF/CD74 axis. A volume of 4‐IPP (80 mg/kg) was diluted in corn oil and subsequently administered by intraperitoneal injection.^31^ Additionally, mice were randomly assigned to be treated with intraperitoneal bolus injections of 4‐IPP (1). The daily dose was 6 mg on the 7th–14th days of treatment.

### 2.19 Survival analysis

The RNA sequencing data and clinical information for cohorts with LSCC were sourced from The Cancer Genome Atlas (TCGA) database (http://xenabrowser.Internet/Information Interface/). The data was used to assess the effect of specific gene combinations on prognosis.

### 2.20 Statistical analysis

In the example, the technique for performing the analytical check is shown. In both images and their description, the numerical value of n is shown exactly, which represents the total number of cells or samples used in the analysis. The GraphPad Prism application from GraphPad Software Inc. was used for data analysis. In the various graphs, examples and additional charts, statistical significance is indicated as follows: An asterisk (*) represents a *p*‐value less than .05, and two asterisks (**) indicate a *p*‐value less than .01. These notations signify a statistically significant difference. A detailed description of other means and materials can be found in the additional information and means section.

## 3 RESULTS

### 3.1 Single‐cell transcriptomic signature of LSCC with COPD

**FIGURE 1:**
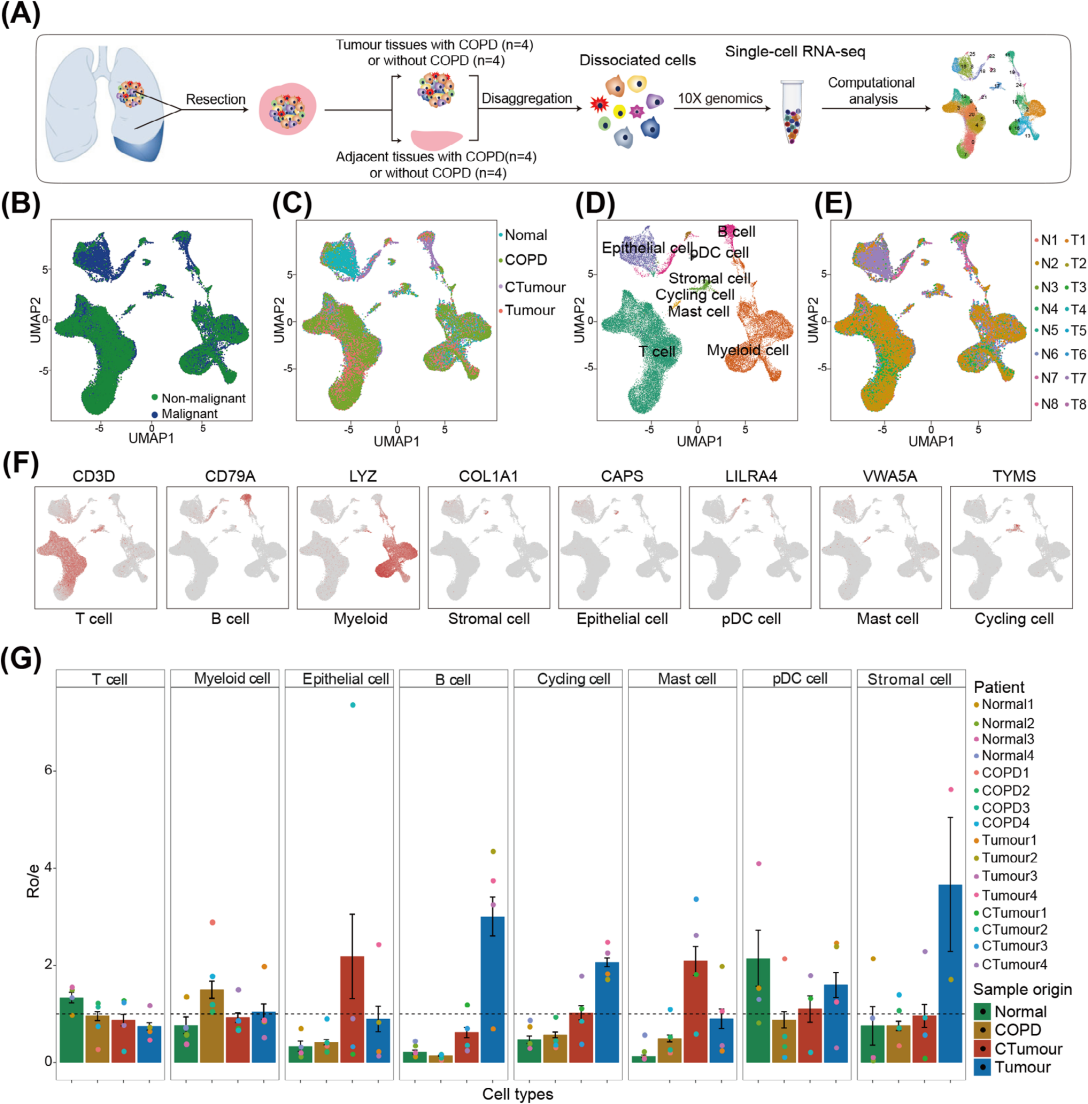
Single‐cell transcriptomic signature of LSCC with COPD: (A) the process of collecting and processing fresh surgical samples from individuals with LSCC (*N* = 4) and LSCC with COPD (*N* = 4), as well as matched normal tissues (*N* = 4) and COPD tissues (*N* = 4) for single‐cell RNA sequencing (scRNA‐seq) analysis using 10× platforms. COPD was classified according to Global Initiative for Chronic Obstructive Lung Disease Staging (GOLD); (B–E) UMAP plots of the 42 462 cells that were color‐coded based on: (B) group origin, (C) main cell types, (D) sample origin and (E) number of transcripts detected (log scale). *K* means thousand; (F) expressions of the key markers used for defining cell types are shown. Additional marker genes can be found Figure S2; (G) relative proportions of the main cell types that were calculated on the basis of the origin of the sample and the group they belong to.

We performed scRNA‐seq (10× Genomics) on eight primary LSCC cases, which included four LSCC without COPD cases (Tumour) and four LSCC with COPD cases (CTumour), as well as adjacent normal tissues (normal) and adjacent COPD tissues (COPD) (Figure 1A; Figure S1A,B). Detailed clinical information of the patients is presented in Table 1 and Table S1.

After quality control, doublet removal, PCA and dimensionality reduction, we identified and partitioned 42 462 high‐quality cells into eight major clusters (Figure 1B, Table S2). Utilizing specific cellular markers,^32^ we categorized the seven identified clusters into immune cells (T cells, B cells, myeloid cells, mast cells and plasmacytoid dendritic cells), epithelial cell and stromal cells (fibroblasts and endothelial cells) (Figure 1C–F; Figure S2A,B; Table S3). The overall cell types observed in both CTumour and Tumour samples were similar (Figure S3A). However, the proportions of lymphoid‐ and myeloid‐derived cells were different (Figure 1G). These differences suggest that the coexistence of COPD may alter the proportions of stromal and immune cells, resulting in a more complex cellular ecosystem in LSCC.

### 3.2 Immunosuppressive roles of TAMs in LSCC with COPD

**FIGURE 2:**
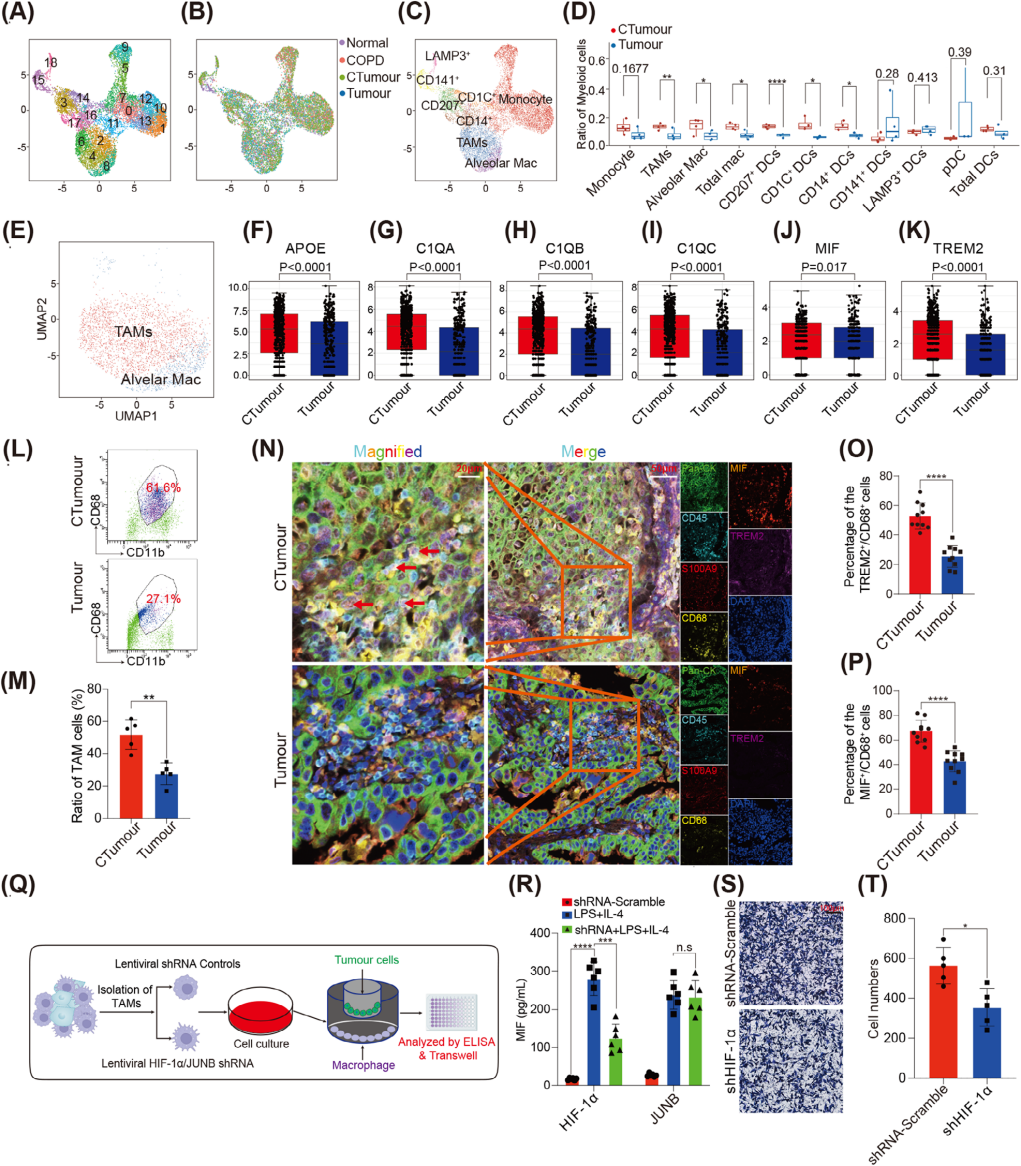
Diversity and functions of myeloid cell lineage in LSCC with COPD: (A–C) UMAP plots of 11 819 myeloid cells color‐coded based on: (A) cluster origin; (B) group origin; (C) main cell types; (D) proportions of different types of myeloid cells, including TAMs, alveolar macrophages (Alveolar Mac), CD141^+^ DCs, CD1C^+^ DCs, CD207^+^ DCs, CD14^+^ DCs, LAMP3^+^ DCs and monocytes were calculated. *p* Value was calculated by paired Wilcoxon test; (E) UMAP plots of main clusters of macrophages; (F–K) expressions of key genes in TAMs were analysed through differential gene expression analysis. *p* value was calculated by Student's test; (L–M) proportions of TAMs and their expression levels of TREM2 were determined by fluorescence‐activated cell sorting (FACS). *p* Value was calculated by Mann–Whitney analysis (*N* = 5); (N) expressions of Pan‐CK, CD45, S100A9, CD68, TREM2 and migration inhibitory factor (MIF) were examined by multiple immunofluorescence (mIF); (O and P) percentages of CD68^+^TREM2^+^ cells and CD68^+^MIF^+^ cells were analysed. Ten pictures were randomly selected for statistics (*N* = 10). *p* Value was calculated by Mann–Whitney analysis; (Q) schematic diagram of the a transwell assay involving NCI‐H520 cells and TAMs treated with shHIF‐α or shJUNB; (R) MIF expressions in TAMs that were quantified with enzyme‐linked immunosorbent assay (ELISA) assays. *p* Value was calculated by two‐way ANOVA test (*N* = 6); (S and T) NCI‐H520 cells were examined using crystal violet staining after co‐culture with TAMs. Five pictures were randomly selected for statistics. *n* = 5. *p < .05, ***p < .001 and ****p < .0001.

We next explored the distinctions among the myeloid cell populations. A total of 19 clusters were identified (Figure 2A,B), including monocytes, macrophages (Mac) and DCs (Figure 2C; Figure S4A–I). We observed a greater proportion of Mac in the CTumour group compared with the Tumour group (Figure 2D,E). Moreover, TAMs in the CTumour group displayed higher levels of APOE, C1QA, C1QB, SPP1, MIF and TREM2 (Figure 2F–K), which may contribute to an immunosuppressive microenvironment.^33^

We isolated tumour‐infiltrating Mac using FACS and validated that the proportion of TAMs was markedly higher in the CTumour group compared with the Tumour group (Figure 2L,M). Furthermore, we observed a notable increase in TREM2 expression levels in the CTumour group, as confirmed by mIF analysis (Figure 2N–P; Figure S5A). In addition, we investigated the correlation between TAMs and patient survival rates using TCGA database. TAMs were identified using a gene signature that included *APOE*, *C1QA*, *C1QB*, *MIF* and *TREM2*. Our analysis revealed that increased TAMs infiltration was associated with poorer overall survival (Figure S5B).

### 3.3 HIF‐1α facilitates the expression of MIF in TAMs

Through SCENIC analysis, we identified significant upregulation of JUNB and HIF‐1α in TAMs from the CTumour group (Figure S5C,D). Notably, previous studies described that HIF‐1α could regulate MIF expression.^31^ We found that MIF was highly expressed in the CTumour group compared with the Tumour group^34^ (Figure 2J). To further investigate how MIF was upregulated in TAMs, we isolated TAMs from CTumour samples using FACS and then stimulated them with IL‐4. Then, JUNB or HIF‐1α was knocked down using lentiviral shJUNB and shHIF‐α (lentiviral shRNAs), respectively (Figure 2Q; Figure S5E). Interestingly, only TAMs treated with shHIF‐1α showed a significant decrease in MIF production (Figure 2R, Figure S5F–I).

Furthermore, in a co‐culture system of NCI‐H520 cells and TAMs, NCI‐H520 cell migration was significantly inhibited when the TAMs were treated with shHIF‐1α compared with those treated with the negative control shRNA (shNC) (Figure 2S,T). These findings indicate that HIF‐1α may promote MIF secretion from TAMs in CTumour samples, thereby enhancing tumour progression.

### 3.4 High expressions of LAMP3, CD80 and CD83 in DCs of LSCC with COPD

As shown in Figure 2D, the proportions of CD207^+^, CD1C^+^ and CD14^+^ DCs were higher in the CTumour group. Additionally, LAMP3^+^ DCs in the CTumour group displayed higher expression levels of CD80, CD83 and LAMP3 (Figure S6A,B). Using unsupervised trajectory analysis, DCs were classified into four categories: classic, migratory, suppressive and mature (Figure S7A,B; Table S4). DCs in the CTumour group were predominantly categorized as classic, whereas those in the Tumour group were mainly distributed in the suppressive stage (Figure S7C).

In the CTumour group, the expression levels of LAMP3, CD80 and CD83 were higher in LAMP3^+^ DCs (Figure S7D,E), whereas CD80 and CD83 were higher in CD207^+^, CD14^+^ and CD1C^+^ DCs, as analysed by FACS (Figure S8A–J). Moreover, mIF staining confirmed that LAMP3 was expressed at higher levels in the CTumour group (Figure S7F–H). Taken together, these findings suggest that LAMP3^+^ DCs, CD207^+^ DCs and CD14^+^ DCs are critical DC clusters in LSCC with COPD.

### 3.5 Increased CD8^+^ T cell exhaustion in LSCC with COPD

**FIGURE 3:**
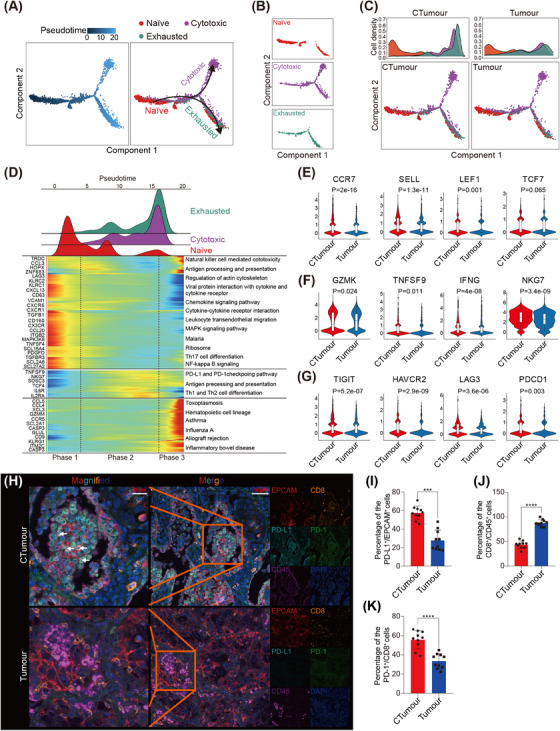
Transition states and expression patterns of CD8^+^ T cell in LSCC with COPD: (A–C) trajectory analysis performed for CD8^+^ T cells, including naïve, cytotoxic and exhausted subsets; (D) heatmap displayed the dynamic variations in gene expression based on trajectory analysis; (E–G) expressions of marker genes for naïve, cytotoxic and exhausted CD8^+^ T cells were assessed. The *p* value was calculated by Student's test; (H) expressions of EPCAM, programmed cell death‐1 ligand 1 (PD‐L1), CD45, CD8 and PD‐1 were examined using multiple immunofluorescence (mIF); (I–K) percentages of EPCAM^+^PD‐L1^+^ cells, CD45^+^CD8^+^ cells and CD45^+^CD8^+^PD‐1^+^ cells were analysed, respectively. Ten pictures were randomly selected for statistics (*N* = 10). The *p* value was calculated by Mann–Whitney analysis. ***p < .001. ****p < .0001.

Of the cell types analysed, T cells were the most prevalent. These included CD8^+^ T cells, CD4^+^ T cells, regulatory T cells (Tregs) and natural killer (NK) cells (Figure S9A–N; Table S5). We then conducted a pseudotime trajectory analysis using Monocles 2 to order each CD8^+^ T cell along a trajectory. We observed a dynamic spectrum spanning from naïve CD8^+^ T cells in the initial state to cytotoxic CD8^+^ T cells in the intermediate state, as well as exhausted CD8^+^ T cells in the terminal state (Figure 3A,B; Table S6). Furthermore, we found that the CD8^+^ T cells in the CTumour group were primarily in the naïve and exhausted states (Figure 3C; Figure S10A). After calculating the expression scores for naïve, cytotoxic and exhausted T cells, we discovered that CD8^+^ T cells in the CTumour group exhibited lower levels of cytotoxic features but higher levels of exhaustion molecules, such as TIGIT, LAG3 and PD‐1 (Figure 3D–G; Figure S11A; Table S7).

To further validate the results of our trajectory analysis, we isolated CD8^+^ T cells using FACS. We observed a significantly higher proportion of exhausted CD8^+^ T cells, as well as higher expression levels of PD‐1 and PD‐L1, in the CTumour group (Figure 3H–K). Furthermore, we identified that both naïve CD8^+^ T cells and exhausted CD8^+^ T cells were significantly more enriched in the CTumour group (Figure S11B,C). Therefore, we concluded that although CD8^+^ T cells in the CTumour and Tumour groups exhibited similar transition trajectories, they demonstrated significant differences in their immune and transcriptional states.

### 3.6 Increased heterogeneity of tumour cells in LSCC with COPD

**FIGURE 4:**
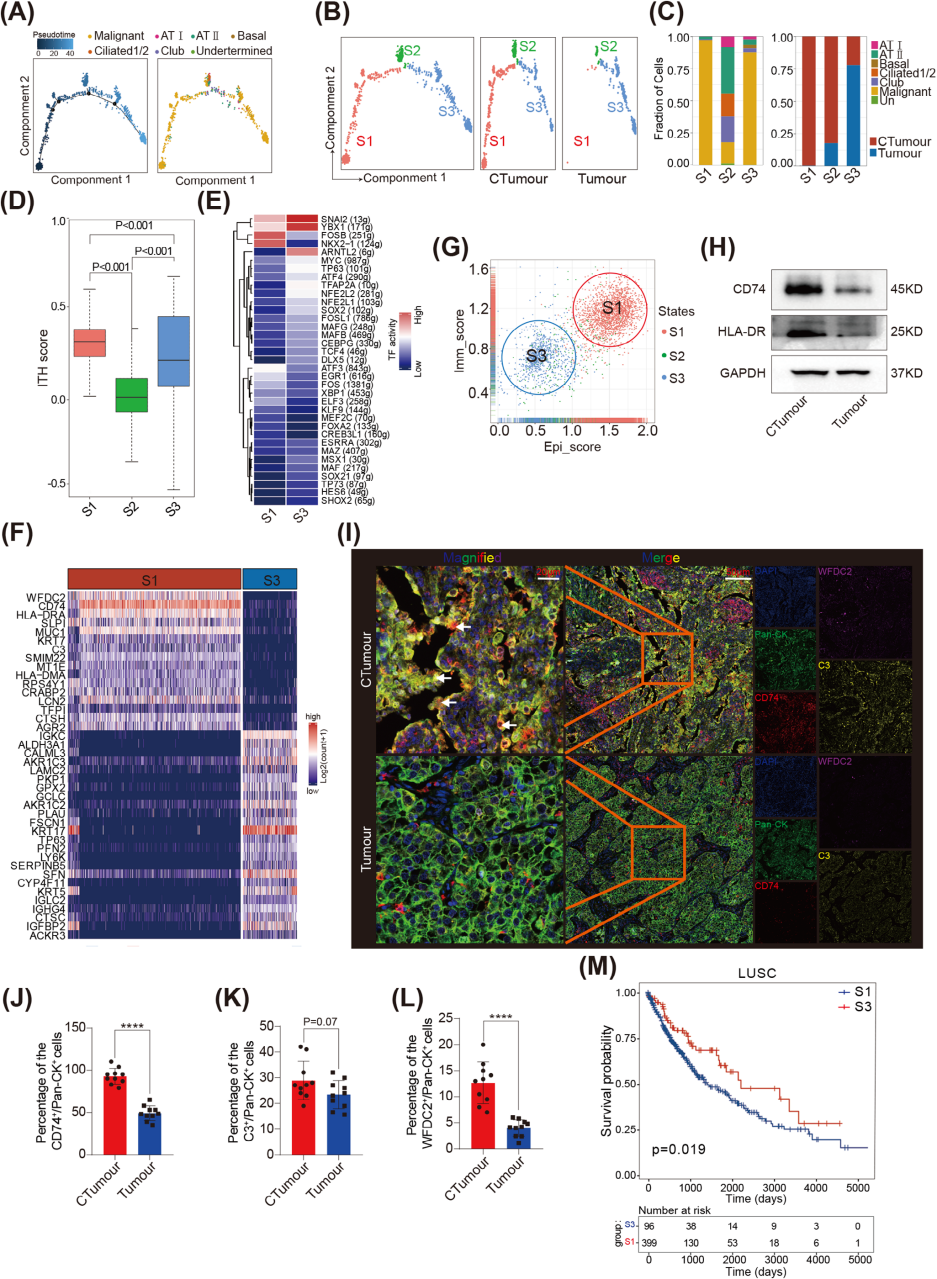
The dual epithelial‐immune features of tumour cells in LSCC with COPD: (A and B) trajectory analysis performed for epithelial cells by utilizing Monocle 2; (C) types and group origins of cells in S1, S2 and S3; (D) **ITH scores of the S1, S2 and S3 subpopulations. The *p* value was calculated by two‐sided Wilcoxon rank‐sum test; (E) the activities of transcription factors in S1 and S3 were analysed using SCENIC analysis; (F) the different patterns of gene expression in S1 and S3 subpopulations; (G) epithelial and immune scores were calculated in the S1, S2 and S3 subpopulations; (H) CD74 and HLA‐DRA were analysed by WB in EPCAM^+^ tumour cells sorted by fluorescence‐activated cell sorting (FACS); (I) expressions of Pan‐CK, CD74, WFDC2 and C3 were examined by multiplex immunofluorescence (mIF); (J–L) percentages of Pan‐CK^+^CD74^+^ cells, Pan‐CK^+^C3^+^ cells and Pan‐CK^+^WFDC2^+^ cells were analysed, respectively. Ten pictures were randomly selected for statistics. *N* = 10. *p* Value was calculated by Mann–Whitney analysis. ****p < .0001; (M) Kaplan–Meier survival analysis was performed for CD74^+^ tumour cells (CD74^+^, HLA‐DRA^+^, HLA‐DMA^+^, MUC1^+^ and WFDC2^+^) in cohorts of lung squamous carcinoma (LUSC) (*N* = 767) from The Cancer Genome Atlas (TCGA). *p* Value was calculated by two‐sided Log‐rank test.

The critical effects of cigarette smoke on lung epithelial cells are associated with the development of COPD and LSCC.^1^ In our studies, we identified a total of 2791 malignant epithelial cells by comparing large‐scale chromosomal CNVs to a reference dataset of normal epithelial cells (Figure S12A–G; Table S8).^35^ By conducting a transcriptional trajectory analysis, the malignant cells could be classified into two opposing branches: S1 and S3. Notably, the S1 subpopulations were primarily derived from the CTumour group, whereas the S3 subpopulation was primarily derived from the Tumour group (Figure 4A‐C). The remaining subpopulation, S2, mostly consisted of normal epithelial cells. AT‐I, AT‐II and basal cells were located at the same branch point along the trajectory, whereas ciliated and club cells were located within a separate branch point (Figure S12H). Additionally, the S1 subpopulation exhibited higher levels of heterogeneity compared with the S2 and S3 subpopulations (Figure 4D).^26^, ^35^

In addition, we identified several key TFs through SCENIC analysis. FOSB and NKX2‐1 showed significant upregulation in the CTumour group compared with the Tumour group (Figure 4E; Figure S13A,B). Previous studies have demonstrated that elevated FOSB and NKX2‐1 expression levels may worsen LC progression in patients with a smoking history.^36^, ^37^ The transcriptional differences between the CTumour and Tumour groups were further validated at the protein level through western blot and IHC assays (Figure S13C,D). Hence, these results suggest that tumour cells in LSCC with COPD exhibit higher heterogeneity and express elevated levels of key TFs that could accelerate LSCC progression.

### 3.7 CD74^+^ tumour cells are correlated with poor survival and aggressive phenotypes

We further assessed the expression features of S1 and S3 subpopulations. We observed that several epithelial–related genes, such as *MUC1* and *KRT7*, were highly expressed in the S1 subpopulation (Figure 4F). Of note, we also observed immune‐related genes in the S1, such as major histocompatibility complex‐II (MHC‐II) genes. Specifically, *CD74*, *HLA‐DRA* and *HLA‐DMA* were identified and listed in Table S9. We observed higher immune scores and a stronger correlation between epithelial scores and immune scores in the S1 subpopulations, indicating that tumour cells in the CTumour group exhibited both epithelial and immune features (Figure 4G; Figure S13E‐J; Table S10). Furthermore, we identified that tumour cells derived from CTumour exhibited higher expression levels of CD74, HLA‐DRA, C3, HLA‐DMA and WFDC2 (Figure S13K‐P). We validated the significant expressions of CD74, WFDC2 and C3 in the CTumour group through western blot and mIF analyses (Figure 4H‐L; Figure S13Q,R).

Subsequently, we analysed a cohort of LSCC from the TCGA and found that a high expression level of CD74^+^ tumour cells was correlated with a worse overall survival (Figure 4M). Furthermore, we performed IF staining for CD74 on lung tumour tissue microarrays (Figure S14A,B). Combining it with the clinical information, we found that a higher expression level of CD74 correlated with worse overall survival (Figure S14C). Collectively, these results demonstrate that CD74^+^ tumour cells are associated with worse prognosis in LSCC.

**FIGURE 5:**
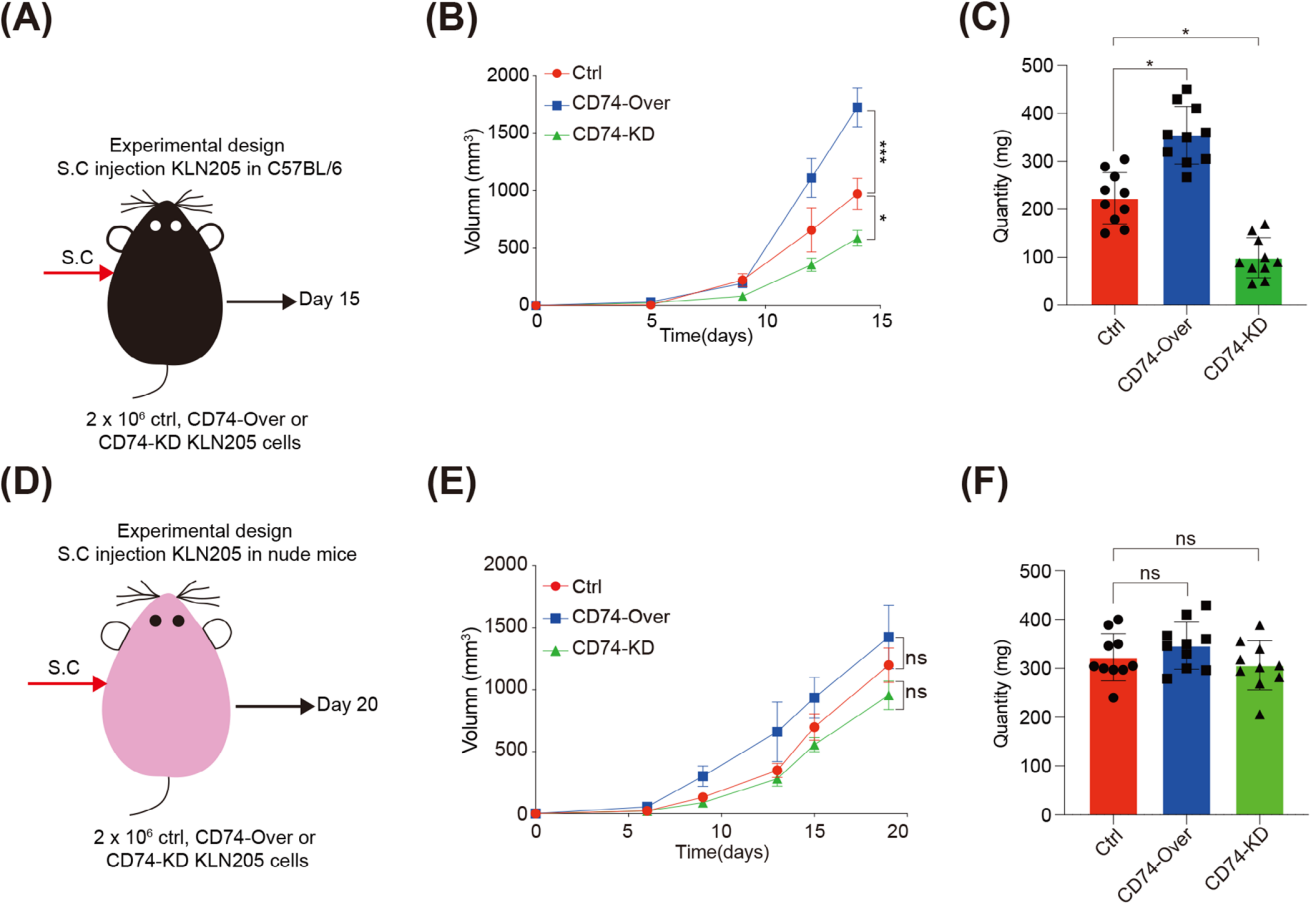
The tumorigenic potential of CD74^+^ tumour cells: (A) tumour growth assay was conducted in C57BL/6 mice after inoculation with CD74‐NC, CD74‐Over and CD74‐KD KLN205 cells; (B) growth curve was generated on the basis of the tumour sizes in C57BL/6 mice measured every 5 days; (C) weights were recorded for the collected tumour tissues from C57BL/6 mice at the 15th day (*N* = 10); (D) tumour growth assay was conducted in nude mice after inoculation with CD74‐NC, CD74‐Over and CD74‐KD KLN205 cells; (E) growth curve was made based on the tumour sizes in nude mice measured every 5 days; (F) weights were recorded for the collected tumour tissues from nude mice at the 20th day (*N* = 10). *p* Value was calculated by one‐way ANOVA test. Data are presented as the mean ± SD. *p < .05, **p < .01 and ***p < .001.

To further investigate the functions of CD74, we generated CD74‐overexpression (CD74‐Over) and CD74‐knock down (CD74‐KD) cells using KLN205 (mouse lung squamous cell cancer line) cells (Figure S15A‐D). We observed that CD74‐Over cells did not exhibit marked proliferation of LC in vitro (Figure S15E‐I). In C57BL/6 mice, the tumour‐forming ability of CD74‐KD KLN205 cells was significantly reduced, as shown in Figures 5A‐C. Furthermore, treatment with the MIF inhibitor 4‐IPP also notably suppressed tumour growth (Figure S15J‐L). However, this effect of CD74‐KD KLN205 cells was not significant in immunodeficient mice (Figure 5D‐F; Figure S15M‐M). These findings suggest that CD74 may facilitate the proliferation of tumour cells, possibly through interactions with immune cells.

### 3.8 CD74 promotes tumour progression by suppressing the cytotoxicity of CD8^+^ T cells

Our study further explored the interactions between tumour and immune cells within the TME. We discovered that monocytes, mac and CD8^+^ T cells were primarily found in CTumour samples. Conversely, Tregs and CD4^+^ T cells were predominantly observed in Tumour samples, as detailed in Figure S16A. CellPhoneDB analysis revealed that the dominant crosstalk in CTumour samples occurred between the S1 subpopulation and myeloid cells, such as TAMs, LAMP3^+^ DCs and CD8^+^ T cells. Of note, the most prominent crosstalk occurred between the S1 subpopulation and exhausted CD8^+^ T cells. Additionally, the interactions between TAMs and exhausted CD8^+^ T cells were predominant in the CTumour group immune cell network (Figure S16B–E). Furthermore, the ligand–receptor pairs between the S1 subpopulation and CD8^+^ T cells, namely CD74‐APP, CD74‐COPA and CD74‐MIF, were significantly involved in cytokine factor signalling in CTumour samples (Figure S17A,B).

**FIGURE 6:**
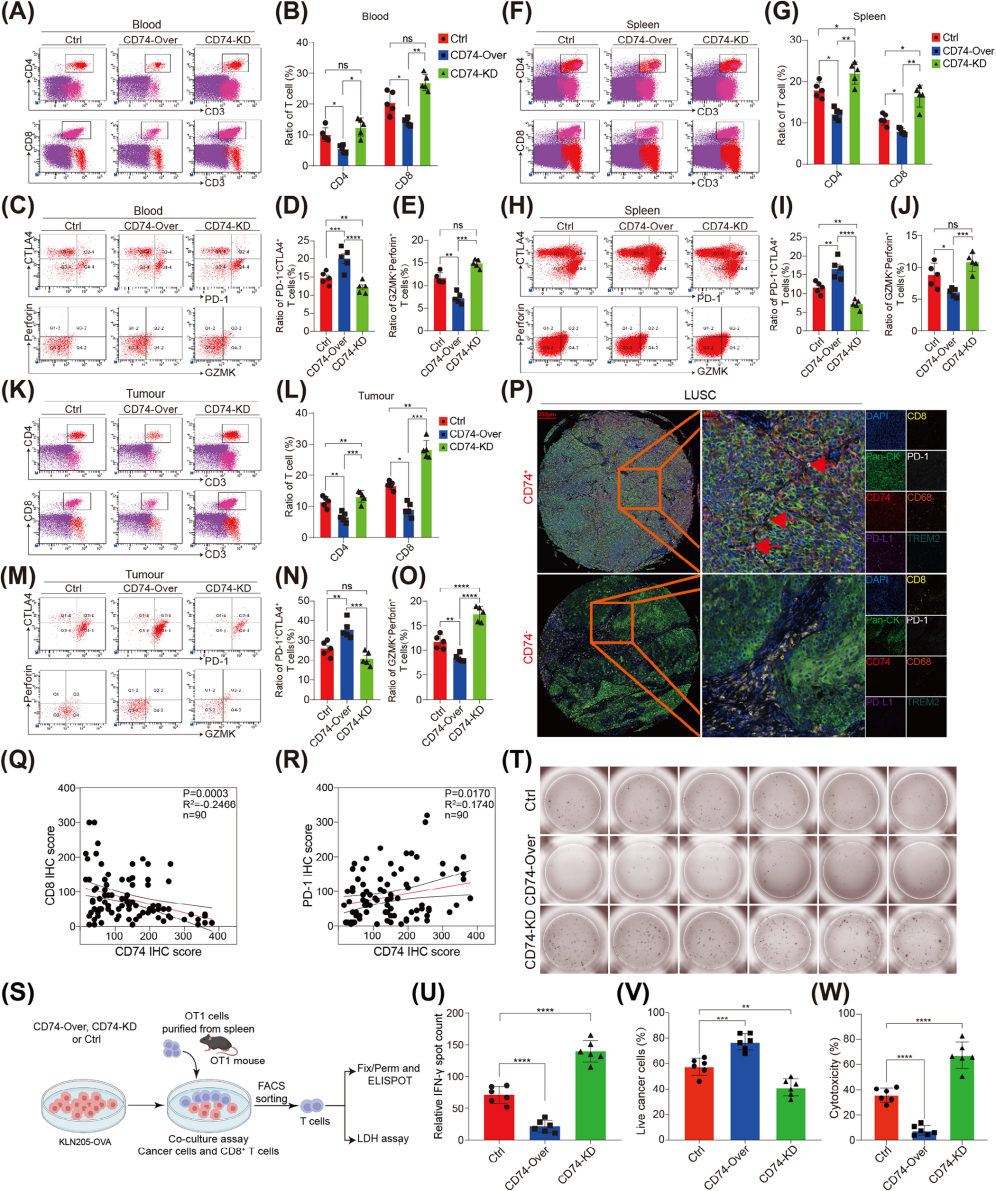
CD74 disruption promotes CD8^+^ T cell‐mediated anti‐tumour immunity: (A, B, F, G, K and L) CD4^+^ T cells (CD45^+^CD3^+^CD4^+^) and CD8^+^ T cells (CD45^+^CD3^+^CD8^+^) were quantified using FACS in mice blood (A and B), spleen (F and G) and tumour (K and L) tissues at 15th day after tumour cells inoculation (*N* = 5); (C, D, H, I, M and N) exhausted CD8^+^ T cells (CD45^+^CD3^+^CD8^+^PD‐1^+^CTLA4^+^) were quantified using FACS in mice blood (C and D), spleen (H and I) and tumour (M and N) tissues at 15th day after tumour cells inoculation (*N* = 5); (C, E, H, J, M and O) cytotoxic CD8^+^ T cells (CD45^+^CD3^+^CD8^+^ GZMK^+^Perforin^+^) were quantified using FACS in mice blood (C and E), spleen (H and J) and tumour (M and O) tissues at 15th day after tumour cells inoculation (*N* = 5). *p* Values were calculated by non‐parametric Dunn's post‐hoc analysis; (P) expressions of CD74, programmed cell death‐1 ligand 1 (PD‐L1), CD8^+^, PD‐1, CD68 and TREM2 were examined by multiple immunofluorescence (mIF) in lung squamous carcinoma (LUSC) (*N* = 90) tissues on microarrays; (Q and R) correlations between CD8 and CD74, as well as PD‐1 and CD74 were analysed in LUSC tissues (*N* = 90) on microarrays; (S) schematic of T cell killing assay using CD8^+^ T cells isolated from OT1 mice and pretreated with OVA peptide, IL‐2 and IL‐7; (T and U) the cytotoxicity of TIL that was measured by ELISPOT assay (*N* = 6). *p* Value was calculated by one‐way ANOVA tests. *p < .05, **p < .01, ***p < .001 and ****p < .0001; (V) live tumour cells isolated by FACS were counted using a Titration assay (*N* = 5). *p* Value was calculated by one‐way ANOVA test; (W) cytotoxicity of CD8^+^ T cells were tested by an LDH assay (*N* = 5). *p* Value was calculated by one‐way ANOVA test. Data are presented as mean ± SD. *p < .05, **p < .01, ***p < .001 and ****p < .0001.

Using FACS analysis, we observed that mice inoculated with CD74‐Over KLN205 cells exhibited a decrease in the relative proportions of CD4^+^ T cells, CD8^+^ T cells and GZMK^+^ Perforin^+^CD8^+^ T cells in the blood, spleen, tumour tissue and lymph nodes. Conversely, there was an increase in the proportion of CD8^+^ T cells expressing PD‐1 and CTLA‐4 (Figure 6A‐O and Figure S18A‐D). Furthermore, our data indicated a higher proportion of terminally exhausted T cells in CD74‐Over tumour tissues. In contrast, the CD74‐KD group showed a higher proportion of progenitor‐exhausted T cells in tumour tissues compared to the Ctrl and CD74‐Over groups (Figures S18E,F and S19A‐I). By employing the mIF methods, we found an inverse relationship between the presence of CD74^+^ tumour cells and CD8^+^ T cells (Figure 6P), as well as a correlation with the ratio of PD‐1 positive CD8^+^ T cells (Figure 6Q,R).

Next, we established a co‐culture assay using mouse spleen CD8^+^ T cells and KLN205 cells (Ctrl, CD74‐Over, CD74‐KD) (Figure 6S). Our findings showed that CD8^+^ T cells co‐cultured with CD74‐Over KLN205 cells produced less interferon (IFN)‐γ compared with T cells co‐cultured with Ctrl‐KLN205 cells (Figure 6T,U). Subsequently, our findings indicated that the cytotoxicity of CD8^+^ T cells was suppressed when co‐cultured with CD74‐Over‐KLN205‐OVA cells (Figure 6V,W). Collectively, these results suggest that CD74 may promote tumour proliferation by suppressing CD8^+^ T cells.

### 3.9 MIF‐CD74 promotes immune evasion by mediating the PI3K‐STAT3‐PD‐L1 signalling pathway

Several studies have demonstrated that high CD74 expression leads to activation of downstream signalling via proto‐oncogene tyrosine‐protein kinases.^38^, ^39^ The MIF‐CD74 signalling axis has been shown to play pivotal roles in initiating an oncogenic signalling pathway.^40^ Importantly, our study demonstrated that MIF secretion by TAMs could enhance tumour progression (Figure 2T).

**FIGURE 7:**
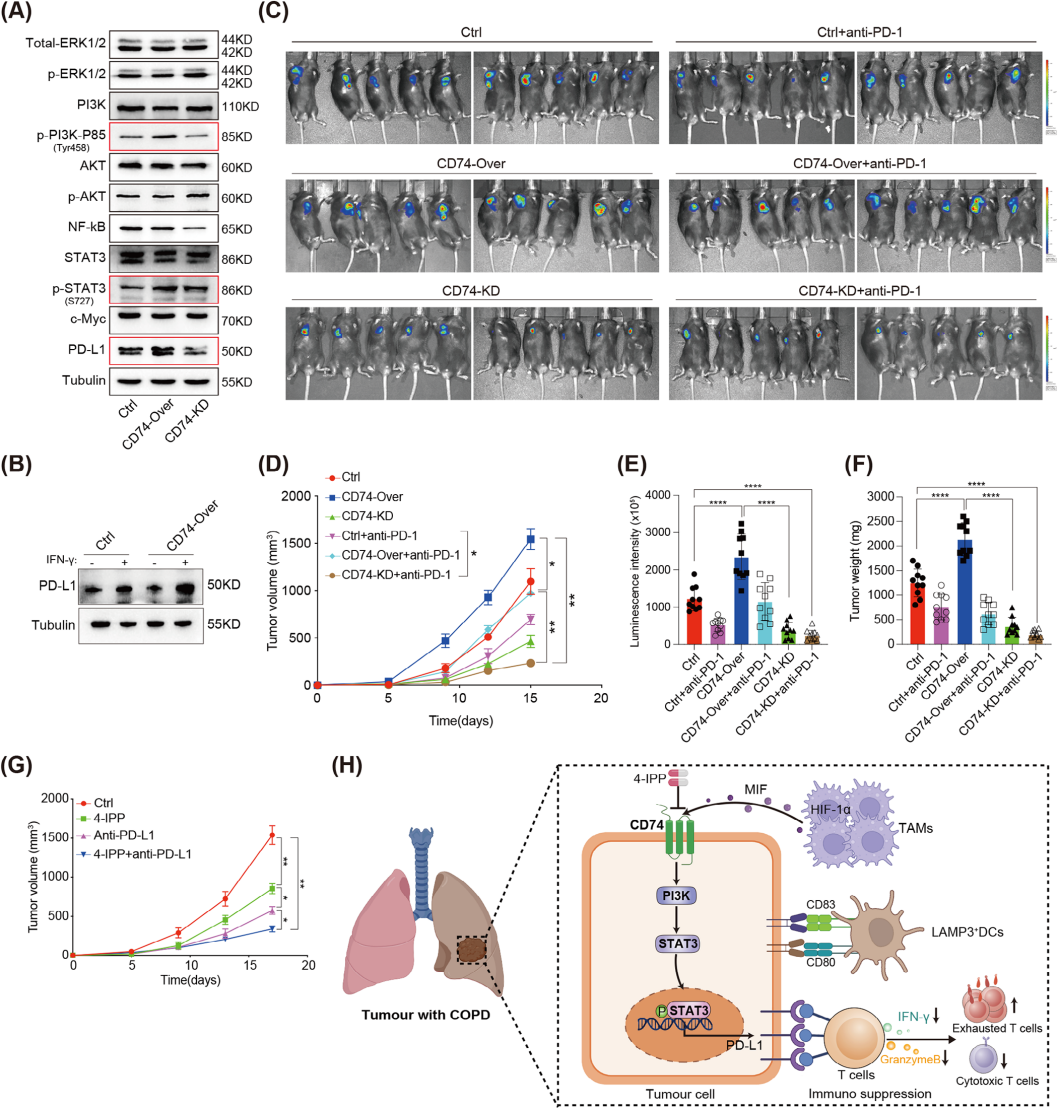
CD74 impairs anti‐tumour immunity by upregulating the PI3K‐STAT3‐PD‐L1 signalling pathway; (A) molecules related to cell proliferation were detected in Ctrl, CD74‐Over and CD74‐KD KLN205 cells by WB assay; (B) KLN205 cell lines were treated with interferon (IFN)‐γ (100 U/mL) for 24 h. PD‐L1 expressions were detected in Ctrl and CD74‐Over KLN205 cells by WB assay; (C–E) growth curve was generated on the basis of the tumour sizes in C57BL/6 mice treated with anti‐PD‐1mAbs, which were measured every 5 days. Luminescence images of tumours were taken in C57BL/6 mice treated with anti‐PD‐1mAbs (on days 8, 10, 12 and 14) at the 15th day after inoculation (*N* = 10). *p* Value was calculated by one‐way ANOVA test; (F) weights were recorded for the tumour tissues from C57BL/6 mice treated with anti‐PD‐1mAbs at the 15th day (*N* = 10). *p* Value was calculated by one‐way ANOVA test; (G) growth curve was generated on the basis of the tumour sizes in C57BL/6 mice treated with anti‐PD‐1mAbs and 4‐IPP inhibitors (*N* = 10); (H) schematic diagram of the mechanisms of CD74‐mediated immunosuppressive effects in lung squamous cell carcinoma (LSCC) with chronic obstructive pulmonary disease (COPD). *p < .05, **p < .01, ***p < .001 and ****p < .0001.

Interestingly, the overexpression of CD74 significantly promoted the phosphorylation of PI3K and STAT3, as well as PD‐L1 expression levels, in KLN205 cells (Figure 7A,B). Indeed, after adding MIF to KLN205 cells, the expression of CD74, phosphorylation of PI3K and STAT3, as well as PD‐L1 significantly increased (Figure S20A–D). However, following CD74 knockdown, PD‐L1 expression levels did not significantly increase (Figure S20E,F). Therefore, these results suggest that the activation of CD74 by MIF may contribute to tumorigenesis by regulating the PI3K‐STAT3‐PD‐L1 signalling pathway.

To restore an effective immune response, we observed that mice receiving a combination of CD74 blockade and an immune checkpoint inhibitor (anti‐PD‐1) exhibited slower tumour growth and improved overall survival (Figure 7C–F; Figure S21A–E). Furthermore, we conducted a therapeutic experiment using a combination of PD‐1 inhibitors and 4‐IPP, a small molecule that prevents MIF from binding to its receptor (CD74). The results showed that this combination significantly reduced tumour growth (Figure 7G; Figure S21F–G). Taken together, TAM‐derived MIF may promote CD74 activation, thus facilitating the PI3K‐STAT3‐PD‐L1 signalling pathway, ultimately resulting in immune escape and tumour progression (Figure 7H).

## 4 DISCUSSION

Our findings from this study helped decipher the presence of a complex immune ecosystem in LSCC with COPD. This ecosystem is characterized by higher levels of CD8^+^ T cell exhaustion molecules and increased proportions of immunosuppressive TAMs, which contribute to the development of an immunosuppressive microenvironment. Further analysis revealed that a critical cluster of CD74^+^ tumour cells, which express both epithelial and immune marker signatures, contributed to immune evasion, ultimately leading to the progression of LSCC with COPD.

Previous studies have found that COPD disrupts the LC immune microenvironment, with CD8^+^ T cells being the most affected cell population.^10^, ^13^ Additionally, PD‐1 expression levels are increased in LC with COPD.^10^, ^13^ Our results demonstrated that CD8^+^ T cells were widely infiltrated in LSCC with COPD samples and exhibited significantly distinct transcriptional features. CD8^+^ T cells from LSCC with COPD were primarily in the naïve and exhausted states, mainly featuring reduced expression levels of cytotoxic factors and increased expression levels of exhaustion molecules, such as TIGIT, LAG3 and PD‐1. Thus, these molecules could functionally impair the CD8^+^ T cells, further facilitating LC progression.^41^ These findings suggest that T cells may transition into an immunosuppressive phenotype during the early premalignant stage of carcinogenesis in the presence of COPD.^13^, ^42^

Recent studies have shown that TREM2^+^ TAMs can facilitate immunosuppression and resistance to immunotherapy, indicating that targeting T cells alone may not be sufficient to overcome immune evasion mechanisms.^33^, ^43^ Our analysis on the myeloid cell milieu revealed a subset of TAMs that was markedly enriched in LSCC with COPD. This subset expressed high levels of TREM2, APOE, C1QA and C1QB and was associated with poorer overall survival in the LSCC patient cohort. Furthermore, we found that MIF was highly expressed in TAMs from LSCC with COPD samples. Through co‐culture experiments, we observed that MIF was regulated by the TF HIF‐1α, which can promote tumour progression.^44^ Our findings demonstrate that TAMs play a crucial role in the TME and may serve as a potential target for immunotherapy in LSCC with COPD. Further investigation is needed to understand how TAMs interact with tumour cells. Additionally, as reported previously, LAMP3^+^ DCs can interact with T cells and NK cells, playing a crucial role in regulating lymphocytes in hepatocellular carcinoma and colorectal cancer.^45^ In our research, we found elevated expression levels of LAMP3, CD80 and CD83 in DCs in LSCC with COPD. This suggests an increased level of DC activation. Therefore, LAMP3^+^ DCs may have a unique ability to interact with T cells, promoting their activation and migration to exert cytotoxic functions.^46^

The mechanisms through which epithelial cells can mediate T cell suppression remain largely unclear.^47^ Through transcriptional trajectory analysis, we discovered a specific cluster of tumour cells in LSCC with COPD that exhibited high expression levels of MHC II‐related genes, including *CD74*, *HLA‐DRA* and *HLA‐DMA*. Although MHC II‐related genes are primarily expressed on antigen‐presenting cells, they can be upregulated in epithelial cells under inflammatory conditions to interact with T cells.^48^ However, the roles of MHC II‐related genes in cancer are controversial. In several types of cancer, the expression patterns of tumour‐specific MHC II‐related genes have been correlated with better outcomes.^49^, ^50^ In melanoma, the high expression of HLA‐DR could impair the effective function of CD8^+^ T cells by inducing the expression of LAG3 and FCRL6.^51^, ^52^ In our study, high CD74 expression in tumour cells exhibited a stronger capacity for tumorigenesis when using normal mouse tumour models.

Previous studies have implicated CD74 as a regulatory factor of cell proliferation and metastasis in a variety of human cancers.^53^, ^54^ Interestingly, by establishing LC cell lines with overexpression or knockdown of CD74, we validated the immunosuppressive roles of CD74 using both a co‐culture system and in vivo experiments. The inhibition of tumour growth by CD74 knockdown was observed solely in C57BL/6 mice, but not in immunodeficient mice, suggesting that CD74 may mediate its effects through interactions with T cells. Of note, Cao et al. recently demonstrated that tumour necrosis factor (TNF)‐α‐dependent lung inflammation can facilitate the upregulation of CD74 in tumour cells, promoting the proliferation and migration of LC cell lines.^55^ In addition, numerous studies have shown that CD74 is the primary receptor for MIF.^56^ Our current study suggests that MIF secreted by TAMs from LSCC with COPD may promote CD74 activation. Therefore, these results show that patients with COPD may develop more pronounced inflammation and associated cytokine production, thus facilitating CD74 activation.^55^, ^57^

PD‐L1/PD‐1‐mediated tumour immune escape is a key challenge in current cancer research.^58^ Our study found that the levels of p‐STAT3, p‐PI3K and PD‐L1 proteins in KLN205 cells significantly increased under different concentrations of MIF. These results suggest that in LN205TAM cells, MIF upregulates PD‐L1 by activating the PI3K/STAT3 signalling pathway, thereby promoting immune evasion. PD‐L1 expression can be induced by external stimuli, such as interferon‐γ produced by tumour cells, and intrinsic oncogenic pathways, such as STAT3 and activated EGFR mutations.^59^, ^60^ The phosphorylated STAT3 protein enters the nucleus in a dimerized form and functions as a TF.^61^ Our study found that MIF‐CD74 regulates the expression of PD‐L1 primarily by activating the STAT3 pathway, which aligns with previous reports. In Figure S18, we observed a significant increase in CD74 expression in the cytoplasm and nucleus after MIF stimulation. Studies indicate that MIF stimulation may promote the entry of proteins within CD74 intracellular domain into the nucleus, further promoting STAT3 phosphorylation and PD‐L1 transcription.^48^ These assumptions need further validation.

The MIF‐CD74 signalling pathway is important in the progression and development of various cancers.^38^, ^55^ Previous studies have demonstrated that inhibiting MIF‐CD74 signalling in MACS and dendritic cells can restore the tumour's immune response to melanoma.^62^ Our data revealed that CD74 protein expression is inversely correlated with the aggregation of CD8^+^ T cells and positively correlated with intra‐individual PD‐1 expression. Through in vitro co‐culture experiments and in vivo modelling, we found that the combined use of MIF‐CD74 blockers and immune checkpoint inhibitors significantly enhances therapeutic efficacy. Therefore, our study suggests that CD74 may impair anti‐tumour activity by interacting with CD8^+^ T cells and modulating the PI3K‐STAT3‐PD‐L1 signalling pathway. Future research will further explore how MIF‐CD74 regulates PD‐L1 expression through additional key signalling pathways.

There are several limitations to this study. Our findings identify specific subsets of CD74^+^ tumour cells in LSCC with COPD and preliminarily explore their regulatory mechanisms and roles using CD74‐overexpressing or knockdown cell lines and co‐culture experiments. However, these results have not been validated in mouse models of LC with COPD or knockout mice. Additionally, single‐cell transcriptomics analysis has limitations in characterizing populations with relatively low abundance, particularly certain subtypes of T cells. To address these limitations, future studies should consider utilizing multi‐omics technologies, such as single‐cell TCR analysis and single‐cell surface protein analysis, which may improve the resolution for detecting these rare but significant cell populations. Furthermore, CD74 and CD74^+^ tumour subsets need to be validated and analysed in larger samples of LSCC with COPD. This includes assessing CD74 expression and its correlation to immunotherapy outcomes.

In summary, our work has revealed differences in tumour ecosystems between LSCC with and without COPD and provided a deeper understanding of the mechanisms by which COPD can affect the development and therapeutic response of LSCC (Figure 7H). Our findings indicate that CD74^+^ tumour cells significantly influence immune responses and could represent a viable therapeutic target in LSCC associated with COPD.

## AUTHOR CONTRIBUTIONS

Weimin Li, Denian Wang and Sixiang Li designed the experiments. Denian Wang, Sixiang Li, Chunyan Yu and others performed the WB, IHC, IF and other experiments. Denian Wang and Chunyan Yu analysed the data and the result of IHC, FACS and Multi‐IF. Denian Wang wrote the article and Weimin Li revised the manuscripts. All authors reviewed the results and approved the final version of the article.

## CONFLICT OF INTEREST STATEMENT

No potential conflicts of interest were disclosed by the other authors. All data generated during this study are included in this article and its Supporting Information files.

## FUNDING INFORMATION

National Natural Science Foundation of China, Grant Numbers: 91859203, 92159302 to Weimin Li; National Natural Science Foundation of China,Grant Number: 82102301 to Denian Wang; Natural Science Foundation of Sichuan, Grant Number: 2024NSFSC0732 to Denian Wang.

## ETHICS STATEMENT

This study was approved by the Ethics Committee of Sichuan University of West China Hospital. The informed consent was signed by every participant.

## CONSENT FOR PUBLICATION

We confirm that all authors have approved the manuscript for submission and the content of the manuscript has not been published or submitted for publication elsewhere.

## Supporting information

Supporting Information

Supporting Information

Supporting Information

Supporting Information

Supporting Information

Supporting Information

Supporting Information

Supporting Information

Supporting Information

Supporting Information

Supporting Information

Supporting Information

Supporting Information

Supporting Information

Supporting Information

Supporting Information

Supporting Information

Supporting Information

Supporting Information

Supporting Information

Supporting Information

Supporting Information

Supporting Information

Supporting Information

Supporting Information

Supporting Information

Supporting Information

Supporting Information

Supporting Information

Supporting Information

Supporting Information

Supporting Information

## Data Availability

The data from our study, including the scRNA‐seq results used, have been deposited in the Comprehensive Gene Expression Database GSE194070 (http://www.ncbi.nlm.nih.gov/geo/query/acc.cgi?acc=GSE194070). Any additional details necessary for reanalyzing the data presented in this article are available upon request from the lead contact. For more information and reagent requests, please reach out directly to Weimin Li at weimi003@scu.edu.cn or Denian Wang at wangdenian623@wchscu.edu.cn.
