# Supplementary material for: Single‐cell transcriptome analysis deciphers the CD74‐mediated immune evasion and tumour growth in lung squamous cell carcinoma with chronic obstructive pulmonary disease
Source: Clin Transl Med. 2024 Aug 7;14(8):e1786. doi: 10.1002/ctm2.1786 (PMC11306293; doi:10.1002/ctm2.1786)
Supplement: Supplementary file 1 — Supporting Information [file CTM2-14-e1786-s012.docx]

**Supplementary Results**

**Single-cell transcriptome analysis deciphers the CD74-mediated immune evasion and tumour growth in lung squamous cell carcinoma with chronic obstructive pulmonary disease**

Denian Wang^1,2,3,#,*^, Sixiang Li^2,4,#^, Zhi Yang^5,#^, Chunyan Yu^6,#^, Pengfei Wu^7^, Ying Yang^7^, Rui Zhang^2^, Qingyan Li^7^, Jian Yang^8^, Hongchun Li^9^, Guiyi Ji^10^, Yan Wang^11^, Kang Xie^1^, Yanyan Liu^12^, Kaige Wang^2^, Daxing Zhu^12^, Wengeng Zhang^1^, Dan Liu^2^, Bojiang Chen^1^, Weimin Li^1,2,3*^

**Affiliations:**

1. Precision Medicine Research Center, Precision Medicine Key Laboratory of Sichuan Province, State Key Laboratory of Respiratory Health and Multimorbidity, West China Hospital, Sichuan University, Chengdu, 610041, Sichuan, China.

2. Department of Respiratory and Critical Care Medicine, Frontiers Science Center for Disease-related Molecular Network, West China Hospital, Sichuan University, Chengdu, 610041, Sichuan, China.

3. Research Units of West China, Chinese Academy of Medical Sciences, West China Hospital, Chengdu, 610041, Sichuan, China.

4. Department of Respiratory and Critical Care Medicine, National Clinical Research Center for Respiratory Disease, the First Affiliated Hospital of Guangzhou Medical University, Guangzhou, 511495, Guangdong, China.

5. Department of Nephrology, West China Hospital, Sichuan University, Chengdu, 610041, Sichuan, China.

6. Frontiers Science Center for Disease-related Molecular Network, Laboratory of Omics Technology and Bioinformatics. West China Hospital, Sichuan University, Chengdu, 610041, Sichuan, China.

7. Department of Respiratory Health, Frontiers Science Center for Disease-related Molecular Network, West China Hospital, Sichuan University, Chengdu, 610041, Sichuan, China.

8.Center of Growth, Metabolism, and Aging, Key Laboratory of Bio-Resources and Eco-Environment, College of Life Sciences, Sichuan University, No.29 Wangjiang Road, Chengdu, 610064, Sichuan, China.

9.National Chengdu Center for Safety Evaluation of Drugs, State Key Laboratory of Biotherapy/Collaborative Innovation Center for Biotherapy, West China Hospital, Sichuan University, Chengdu, 610041, Sichuan, China.

10.Health Management Center, West China Hospital, Sichuan University, Chengdu, 610041, Sichuan, China.

11.Department of Thoracic Surgery, West China Hospital, Sichuan University, Chengdu, 610041, Sichuan, China.

12. Lung Cancer Center, West China Hospital, Sichuan University, No. 37 Guoxue Alley, Chengdu, 610041, Sichuan, People's Republic of China.

^#^ These authors contributed equally: Denian Wang, Sixiang Li, Zhi Yang, and Chunyan Yu.

*Weimin Li, MD, Professor. Precision Medicine Research Center, Department of Respiratory and Critical Care Medicine, Frontiers Science Center for Disease-related Molecular Network, West China Hospital, Sichuan University, Chengdu, Sichuan, China. No. 37, Guoxue Alley, Wuhou District, Chengdu, 610041, Sichuan, China. E-mail: [weimi003@scu.edu.cn](mailto:weimi003@scu.edu.cn).

*Denian Wang, Professor. Precision Medicine Research Center, Precision Medicine Key Laboratory of Sichuan Province, State Key Laboratory of Respiratory Health and Multimorbidity, West China Hospital, Sichuan University, Chengdu, Sichuan, China. No. 37, Guoxue Alley, Wuhou District, Chengdu, 610041, Sichuan, China. E-mail: wangdenian623@wchscu.edu.cn.

Tel: +86-29-85423998;

Fax: +86-28-85582944;

Ethics approval and consent to participate

This study was approved by the Ethics Committee of Sichuan University of West China Hospital. The informed consent form was signed by every participant.

**Supplemental methods**

**Cell culture**

The THP-1 (RRID: CVCL_0006), NCI-H520 (human lung squamous cell cancer line, RRID: CVCL_1566) and KLN205 (mouse lung squamous cell cancer line, RRID: CVCL_3533) cell lines were purchased from Cell Bank/Stem Cell Bank Chinese Academy of Science (China) and cultured in Dulbecco's modified Eagle’s medium (DMEM; Gibco), supplemented with 10% fetal bovine serum (FBS; Gibco) and 1% penicillin-streptomycin. The cells were maintained following ATCC guidelines at 37°C in a 5% CO_2_ environment. Cell growth was monitored. To induce differentiation of THP-1 into macrophages, we added phorbol 12-myristate 13-acetate (PMA) (200 ng/mL) to the medium and cultured it for 48 hours. Subsequently, we cultured it with fresh medium without PMA for an additional 24 hours. In addition, all cell lines (THP-1, NCI-H520, and KLN205) were tested for mycoplasma contamination prior to experimentation. The cell lines used are free from mycoplasma contamination.

Murine cell lines overexpressing CD74 (CD74-Over) were established using lentiviral transduction with the pSLenti-EF1-CD74-EGFP-P2A-Puro-CMV-MCS-3xFlag vector (OBiO, China). Murine cell lines with CD74 knockdown (CD74-KD) were established using lentiviral transduction with the pSLenti-U6-shRNA-CMV-F2A-Puro-WPRE lentiviral vector (OBiO, China). KLN205-OVA cell lines were established by lentiviral transduction using the pSLenti-EF1-mCherry-P2A-Puro-CMV-OVAL-3xFLAG-WPRE vector (OBiO, China).

**Western blotting (WB) analysis**

The WB analysis was performed as previously described^1^. The antibodies utilized were as follows: FOSB (Cell Signaling Technology, #2263, 1:1000), NKX2-2 (Cell Signaling Technology, #12310, 1:1000), CD74 (Cell Signaling Technology, #77274T, 1:1000), HLA-DR (Cell Signaling Technology, #43816, 1:1000), PI3K(Cell Signaling Technology, #4255S, 1:1000), p-PI3K (Cell Signaling Technology, #4288S, 1:1000), ERK1/2 (Cell Signaling Technology, #4695S, 1:1000), p-ERK1/2 (Cell Signaling Technology, #4370S, 1:1000), c-Myc (Cell Signaling Technology, #5605S, 1:1000), PD-L1 (Abcam, #ab213524, 1:1000), STAT3 (Cell Signaling Technology, #9193S, 1:1000), p-STAT3 (Abcam, #9145S, 1:1000), AKT (Cell Signaling Technology, #4685S, 1:1000), p-AKT (Cell Signaling Technology, #4046S, 1:1000), NF-kB (Cell Signaling Technology, #8542S, 1:1000), Actin (Cell Signaling Technology, #3700S, 1:1000), HRP-linked anti-rabbit IgG (Cell Signaling Technology, #7074S, 1:3000), and HRP-linked anti-mouse IgG (Cell Signaling Technology, #7076S, 1:3000).

**Immunohistochemistry (IHC)**

The IHC analysis was performed as described previously^1^. The antibodies used were as follows: anti-FOSB (rabbit, 1:200, Cell Signaling Technology, Cat# 2251T), anti-NKX2.1 (rabbit, 1:200, R&D, MAB8187-SP), anti-CD74 (rabbit, 1:200, Cell Signaling Technology, Cat# 77274T), and anti-HLA-DR (rabbit, 1:200, Abcam, Cat# ab92511).

**Multiple immunofluorescence staining**

Multiple staining was performed using the Opal Polaris 7-Color Manual IHC Kit (NEL861001KT) following the manufacturer's instructions. The HRP-conjugated secondary antibody polymer was detected using fluorescent signal amplification with Opal dyes 480, 520, 570, 620, 690, and 780. For macrophages, a panel comprising CD45, S100A9, CD68, SPP1, and TREM2 was employed to label TAMs and TREM2 in TAMs. CD45 represents leukocytes, S100A9 represents myeloid cells, and CD68 represents macrophages. To label LAMP3^+^ DCs, we employed a panel comprising CD45, HLA-DR, LAMP3. For T cells, to label exhausted CD8^+^ T cells and PD-1 expression in exhausted CD8^+^ T cells, we utilized a panel consisting of CD45, CD8, PD-1, EPCAM, and PD-L1.

The antibodies utilized were as follows: CD45 (Abcam, Cat# ab40763, 1:100), CD3 (Abcam, Cat# ab16669, 1:100), CD8 (Abcam, Cat# ab237709, 1:100), PD-L1 (Abcam, Cat# ab213524, 1:50), EPCAM (Abcam, Cat# ab213550, 1:50), CD63 (Abcam, Cat# ab271286, 1:50), SPP1 (Abcam, Cat# ab63856, 1:50), TREM2 (Abcam, Cat# ab223684, 1:50), PD-1 (Abcam, Cat# ab52587, 1:50), CD68 (Abcam, Cat# ab213363, 1:50), S100A9 (Abcam, Cat# ab22506, 1:50), CD74 (Abcam, Cat# ab270265, 1:100), C3 (Abcam, Cat# ab200999, 1:100), WFDC2 (Abcam, Cat# ab200828, 1:100), Pan-CK (Abcam, Cat# ab7753, 1:50), MIF (Abcam, Cat# ab227073, 1:100) and HLA-DR (Abcam, Cat# ab92511, 1:100).

**Real-time PCR**

The RT-PCR analysis was performed as previously described^2^. The primer sequences are as follows:

FOSB: (F) GCTTCTCTCTTTACACACAGTG; (R) TGAGGACAAACGAAGAAGTGTA;

NKX2.1: (F) TTCGGTCAAGGACATCTTAGAC; (R) CTGTCGTAGAAGGGGTTCTT;

HIF-1α: (F)TGCAACATGGAAGGTATTGC-3; (R)TTCACAAATCAGCACCAAGC-3’;

**Flow cytometry analysis**

The flow cytometry analysis was performed as previously described^1^. To investigate the functions of T cells, we stained the cells using the following antibodies: anti-CD45 (CD45-PE, BD Pharmingen, Cat# 555483), anti-CD279/PD-1 (CD279-BV605, BD Pharmingen, Cat# 563245), anti-CD3 (CD3-BV510, BD Pharmingen, Cat# 566779), anti-CD45RA (CD45RA-BV786, BD Pharmingen, Cat# 563870), anti-CCR7 (CCR7-PE-Cy7, BD Pharmingen, Cat# 557648), anti-Granzyme (Granzyme-R718, BD Pharmingen, Cat# 566964), anti-Perforin (Perforin-BV421, BD Pharmingen, Cat# 563393), anti-CD152 (CD152-BB515, BD Pharmingen, Cat# 566917) and anti-CD8 (CD8-APC, BD Pharmingen, Cat# 340659). The T cells were fixed and permeabilized using Intracellular Staining Perm Wash Buffer (BD Pharmingen, Cat# 562574).

To examine TAMs, we stained the cells using the following antibodies: anti-CD45 (CD45-PE, BD Pharmingen, Cat# 555483), anti-CD11b (CD11b-FITC, Biolegend, Cat# 301330) and anti-CD68 (CD68-PE-Cy7Cy7, BD Pharmingen, Cat# 565595). DCs were identified using the following antibodies: anti-CD45 (CD45-PE, BD Pharmingen, Cat# 555483), anti-CD63 (CD63-BV786, BD Pharmingen, Cat# 741004), anti-CD141 (CD141-BV421, BD Pharmingen, Cat# 565321), anti-CD123 (CD123-R718, BD Pharmingen, Cat# 752032), anti-CD1C (CD1C-APC-R700, BD Pharmingen, Cat# 566614), anti-CD14 (CD14-BV750, BD Pharmingen, Cat# 746920), anti-CD80 (CD80-PerCP-Cy5.5, BD Pharmingen, Cat# 567436), anti-CD83 (CD83-PE-Cy5, BD Pharmingen, Cat# 551058), anti-CD207 (CD207-PE-Cy7, Biolegend, Cat# 144209), and anti-CD86 (CD86-PE-Cy5, BD Pharmingen, Cat# 555666).

Brefaldin A (250 μg; Sigma-Aldrich, Cat# B7651) was used for intracellular staining. Tumour tissues were digested at 37°C for 30-45 min with 1 mg/mL Collagenase Ⅰ, 0.5 mg/mL Collagenase ⅠV, and 0.1mg/mL DNase Ⅰ (Roche). Cell suspensions were filtered through a 70-μm filter and resuspended in staining buffer with following antibodies for 30 min: anti-CD45 (CD45-FITC, BD Pharmingen, Cat# 561088), anti-CD3 (CD3-Per-cy5.5, BD Pharmingen, Cat# 551163), anti-CD8 (CD8-APC-Cy7, BD Pharmingen, Cat# 557654), anti-CD4 (CD4-BV510, BD Pharmingen, Cat# 563106), anti-CCR7 (CCR7-BV421, BD Pharmingen, Cat# 562675), anti-CD45RA (CD45RA-BV711, BD Pharmingen, Cat# 740681), anti-GZMK (GZMK-PE, Thermo, Cat# 12-8898-82), anti-Perforin (Perforin-APC, Thermo, Cat# 17-9392-80), anti-CTLA-4 (CTLA-4-R700, BD Pharmingen, Cat# 565778), anti-PD-1 (PD-1-BV605, BD Pharmingen, Cat# 563059), anti-IFN-γ (IFN-γ-PE-Cy7, BD Pharmingen, Cat# 561040).

**Cell counting kit-8 (CCK-8) assay**

KLN205-Ctrl, KLN205-Over, and KLN205-KD cells were seeded in 96-well plates and cultured with serum-free medium for 24, 48, 72, 96, or 120 hours. The medium was replaced, and 10 μL of CCK-8 was added to each well. The cells were then incubated at 37°C with 5% CO_2_ for 1 to 4 hours. The optical density (OD) values were measured at a wavelength of 450 nm to detect the proliferation activity of cells.

**Colony formation assay**

KLN205-Ctrl, KLN205-Over, and KLN205-KD cells were seeded in 6-well plates and cultured for 10 days. At the end of the incubation period, the cells were washed twice with FBS, fixed in methanol, and stained with crystal violet.

**Migration assay**

NCI-H520-Ctrl, NCI-H520-shHIF-1α (GIPZ Lentiviral Human HIF-1α shRNA, Cat# V2LMM_69359, Dharmacon) and NCI-H520-shJUNB (GIPZ Lentiviral Human JUNB shRNA, Cat# V2LHS_133575, Dharmacon) cells were seeded in 24-well plates. After 48 hours, non-migratory cells were removed using a cotton swab infused with 70% ethanol. The migratory cells were fixed in methanol, stained with crystal violet, and counted.

**Animals**

The male C57BL/6 mice (five-week-old) and nude mice (five-week-old) were obtained from the animal center of Beijing Huafukang. OT1 mice (six-week-old) were purchased from Model Animal Research Center of Nanjing University and Gempharmatech Company. The animals were kept in a specific pathogen-free environment with controlled temperature, following a 12-hour light/12-hour dark cycle. They were provided with free access to food and water. All animal experiments were conducted in accordance with institutional and national guidelines and approved by the Animal Ethics Committee of Sichuan University. In accordance with animal welfare guidelines, mice with subcutaneous tumours exceeding 1500 mm^3^ should be euthanized.

**Supplemental Figure legends**

**Figure legends**

**Figure S1.** **A-B,** The UMAP plot of 42,462 cells that are color-coded based on (A) clusters; (B) group origin.

**Figure S2. A-B,** Expressions of marker genes that were used for defining cell types.

**Figure S3.** **A,** The data of the 16 epithelial subclusters, 67 immune cell (T cells, B cells, myeloid cells, mast cells, pDCs) subclusters, 5 stromal cells (fibroblast and endothelial cells) subclusters, and 10 cycling cell subclusters of 42,462 cells from 16 samples (Left to right): the fraction of cells originating from the 8 non-malignant samples and 8 tumour samples, the fraction of cells originating from each sample of the 8 patients, number of cells, and box plots of the number of transcripts.

**Figure S4. A-I,** Violin plots showing the expressions of the key markers used for defining myeloid cells. **J,** UMAP plot showing the expressions of the key markers used for defining myeloid cells.

**Figure S5. A,** Expressions of Pan-CK, CD45, S100A9, CD68, TREM2 and MIF were examined by multiple immunofluorescence (mIF) in Normal and COPD samples. **B,** Kaplan-Meier survival analysis for TAMs (*APOE*, *C1QA*, *C1QB*, *MIF*, *TREM2*) in cohorts of lung squamous carcinoma (LUSC) (N=767) from The Cancer Genome Atlas (TCGA). *P* value was calculated by two-sided Log-rank test. **C-D,** The activities of transcription factors in TAMs detected by using SCENIC analysis. **E,** The NCI-H520 cells were transfected with the shRNA candidates of HIF-1α and JUNB (using lentiviral particles). The mRNA level of HIF-1α and JUNB was measured by Real-time RT-PCR. **F-I,** The expression of MIF in NCI-H520, KLN205, THP-1, and TAMs after 24 and 48 hours of hypoxia was detected using ELISA. *P* values were calculated by one-way ANOVA tests. **P* < 0.05, ***P* <0.01, ****P*<0.001.

**Figure S6. A-B,** Median expressions of marker genes were analyzed for DC subsets. The *P* value was calculated by Tukey’s multiple comparison analysis.

**Figure S7. A-C,** Trajectory analyses that were performed for DCs, including classic DCs, migratory DCs, suppressive DCs and mature DCs. **D-E,** Proportions of LAMP3^+^ DCs and their expression levels of LAMP3, CD80, CD83 and CD86 were tested by FACS. The *P* value was calculated by Sidak’s multiple comparisons test. **F-H,** Expressions of CD45, HLA-DR and LAMP3 (CD63) were examined by mIF. Ten pictures were randomly selected for statistics (N=10). *P* value was calculated by Mann-Whitney analysis. **P* < 0.05, ** *P* < 0.01.*** *P* < 0.001.

**Figure S8. A-B,** Proportions of CD1C^+^ DCs and the expression levels of CD80, CD83, and CD86 in CD1C^+^ DCs were measured by FACS. **C-D,** Proportions of CD14^+^ DCs and the expression levels of CD80, CD83, and CD86 in CD14^+^ DCs were measured by FACS. **E-F,** Proportions of pDCs and the expression levels of CD80, CD83, and CD86 in pDCs were measured by FACS. **G-H,** Proportions of CD141^+^ DCs and the expression levels of CD80, CD83, and CD86 in CD141^+^ DCs were measured by FACS. **I-J,** Proportions of CD207^+^ DCs and the expression levels of CD80, CD83, and CD207^+^ DCs were measured by FACS. The *P* value was calculated by Sidak’s multiple comparisons test. ns, not significant, **P* < 0.05, ***P* <0.01, ****P*<0.001, *****P*<0.0001.

**Figure S9. A-C,** UMAP plot of the 22,835 cells that were color-coded based on: (A) clusters; (B) group origin; (C) T cells subtypes. **D,** Proportions of different types of T cells. **E-L,** Expressions of the key markers that were used for defining T cell types. **M,** UMAP plot of T cells that were color-coded based on subtypes, including natural killer cells (NK), CD8^+^ naïve T cells (CD8_na_), CD8^+^ cytotoxic T cells (CD8_cyt_), CD8^+^ effective T cells (CD8_eff_), CD8^+^ exhausted T cells (CD8_exh_), tissue resident memory CD8^+^ T cells (T_RM_), Th2, Th17, Tfh, Treg, tissue resident memory CD4^+^ T cells (T_RM_), naïve CD4^+^ T cells (CD4_na_). **N,** Proportions of different subtypes of T cells.

**Figure S10. A,** Heatmap showing the expression levels of marker genes specific to T cells and NK cells.

**Figure S11. A,** Expressions of functional genes associated with naivety, cytotoxicity, and exhaustion along the developmental trajectory of CD8^+^ T cells. **B-C,** Proportions of naïve T cells, cytotoxic T cells and exhausted T cells were measured by FACS. *P* values were calculated by Sidak’s multiple comparisons test. **P* < 0.05, ***P* <0.01, ****P*<0.001, *****P*<0.0001.

**Figure S12. A-B,** UMAP plot of the 3,758 epithelial cells that were color-coded based on: (A) clusters; (B) group origin; **C,** Proportions of different types of epithelial cells. **D,** UMAP plot of epithelial cells subsets. **E-F,** Expressions of the key markers that were used for defining epithelial cells. **G,** CNVs analysis performed for epithelial cells. **H,** Trajectory analysis of epithelial cells conducted by using Monocle 2.

**Figure S13. A-C,** Relative mRNA and protein levels of FOSB and NKX2-1. *P* values were calculated by Mann–Whitney test (N=4). **D,** Expression levels of FOSB that were detected by IHC staining (N=10). **E**, Correlations between the S1 and S3 subpopulations were examined according to Pearson residuals. **F-J**, Co-expression of EPCAM and immune-related genes. **K-P**, Expressions of immune-related genes and epithelial-related genes through differential gene expression analysis. **Q**, Expression levels of CD74 that were detected by IHC staining. **R**, Relative expression scores of CD74 (N=10). *P* values were calculated by one-way ANOVA tests. **P* < 0.05, ***P* <0.01, ****P*<0.001.

**Figure S14**. **A,** Expressions of CD74 were analyzed in LUSC tissues (N=90) and adjacent normal tissues (N=90) on microarrays. **B,** The CD74 expression scores. *P* values were calculated by Mann-Whitney test. **C**, Kaplan-Meier survival analysis for expression level of CD74 (CD74^high^ or CD74^low^) in the cohort of LUSC. *P* value was calculated by two-sided Log-rank test.

**Figure S15. A-B,** Overexpression of CD74 in KLN205 cells was verified by WB assay. **C-D,** Knockdown of CD74 in KLN205 cells was verified by WB assay. **E,** Ability of cell proliferation was evaluated in KLN205 cells under the influence of CD74 using the CCK-8 assay. *P* values were calculated by Sidak’s multiple comparisons test. **F-G,** Ability of cell proliferation was evaluated in KLN205 cells under the influence of CD74 using the clone formation assay. *P* values were calculated by one-way ANOVA test. **H-I,** Ability of cell proliferation was evaluated in KLN205 cells under the influence of CD74 using IF staining. *P* values were calculated by one-way ANOVA test. ns, not significant, **P* < 0.05, ***P* <0.01, ****P*<0.001, *****P*<0.0001. **J,** Images were recorded for the collected tumour tissues from nude mice at the 20th day (n=5). **K,** Growth curve was generated based on the tumour sizes in C57BL/6 mice measured every 5 days. **L.** Weights were recorded for the collected tumour tissues from C57BL/6 mice at the 15th day (N=5). **M-N,** CD3^+^ T cells were quantified using FACS in nude and C57BL/6 mice blood (N=5).

**Figure S16. A,** The distribution map of 29 cell subtypes in NSCLC with COPD according to Pearson residuals. **B-E,** Significant cell-to-cell interactions were displayed in heatmaps.

**Figure S17. A,** Selected ligand-receptor interactions between tumour cells and immune cells. **B,** Selected ligand-receptor interactions between CD8^+^ T cells and macrophages.

**Figure S18. A and B,** CD4^+^ T cells (CD45^+^CD3^+^CD4^+^) and CD8^+^ T cells (CD45^+^CD3^+^CD8^+^) were quantified using FACS in mice lymph nodes tissues at 15^th^ day after tumour cells inoculation (N=3). **A and C**, Cytotoxic CD8^+^ T cells (CD45^+^CD3^+^CD8^+^ GZMK^+^Perforin^+^) were quantified using FACS in mice lymph nodes tissues at 15^th^ day after tumour cells inoculation (N=3). **A and D,** Exhausted CD8^+^ T cells (CD45^+^CD3^+^CD8^+^PD-1^+^CTLA4^+^) were quantified using FACS in mice mice lymph nodes tissues at 15^th^ day after tumour cells inoculation (N=3). **A and E, and D,** Ly108^+^exhausted CD8^+^ T cells (CD45^+^CD3^+^CD8^+^PD-1^+^CTLA4^+^Ly108^+^) were quantified using FACS in mice mice lymph nodes tissues at 15^th^ day after tumour cells inoculation (N=3). **A and F,** Tim-3^+^ exhausted CD8^+^ T cells (CD45^+^CD3^+^CD8^+^PD-1^+^CTLA4^+^Tim-3^+^) were quantified using FACS in mice mice lymph nodes tissues at 15^th^ day after tumour cells inoculation (N=3). *P* values were calculated by non-parametric Dunn’s post-hoc analysis.

**Figure S19.** **A-I,** Ly108^+^ exhausted CD8^+^ T cells and Tim-3^+^ exhausted CD8^+^ T cells were quantified using FACS in mice blood (A-C), spleen (D-F) and tumour (G-I) tissues at 15^th^ day after tumour cells inoculation (N=5).

**Figure S20. A-D**, KLN205 cell lines were treated with MIF (25μg/mL or 50μg/mL) for 24h, respectively. Molecules related to cell proliferation were detected in KLN205 cells by WB assay and immunofluorescence. **E-F,** KLN205 cell lines were treated with MIF (50μg/mL) for 24h. PD-L1 expression were detected in Ctrl, CD74-Over KLN205, and CD74-KD cells by immunofluorescence.

**Figure S21.** **A-C,** Representative images of H&E, CD8, and PD-L1 staining for tumour tissues collected from C57BL/6 mice treated with anti-PD-1 mAbs on the 15th day. **D and E,** The relative expression of CD8 and PD-L1 based on fluorescence density. **F and G,** Representative images of PD-L1 staining for tumour tissues collected from C57BL/6 mice treated with the MIF inhibitor (4-IPP).

**Reference:**

1. Xiao, F., Wang, D., Kong, L., Li, M., Feng, Z., Shuai, B., Wang, L., Wei, Y., Li, H., Wu, S., et al. (2018). Intermedin protects against sepsis by concurrently re-establishing the endothelial barrier and alleviating inflammatory responses. Nat Commun *9*, 2644. 10.1038/s41467-018-05062-2.

2. Wang, L.J., Xiao, F., Kong, L.M., Wang, D.N., Li, H.Y., Wei, Y.G., Tan, C., Zhao, H., Zhang, T., Cao, G.Q., et al. (2018). Intermedin Enlarges the Vascular Lumen by Inducing the Quiescent Endothelial Cell Proliferation. Arterioscler Thromb Vasc Biol *38*, 398-413. 10.1161/atvbaha.117.310317.
