# Supplementary figures and images for: Single‐cell transcriptome analysis deciphers the CD74‐mediated immune evasion and tumour growth in lung squamous cell carcinoma with chronic obstructive pulmonary disease
Source: Clin Transl Med. 2024 Aug 7;14(8):e1786. doi: 10.1002/ctm2.1786 (PMC11306293; doi:10.1002/ctm2.1786)

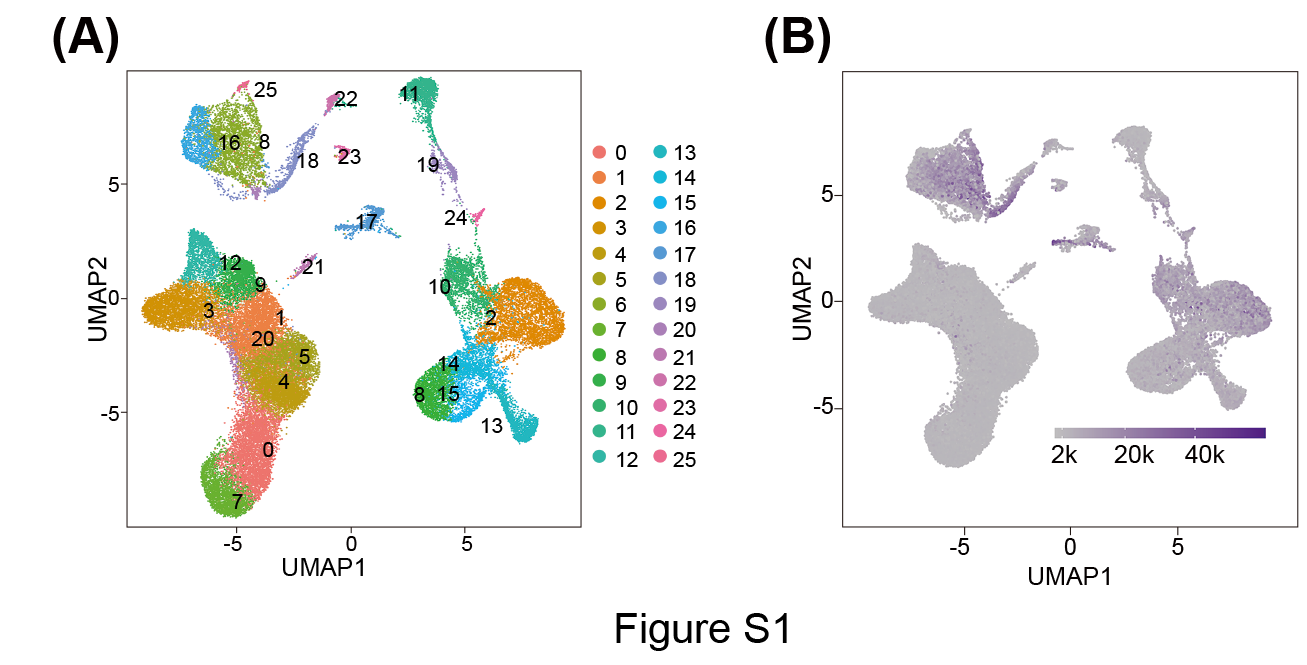

Supplement: Supplementary file 2 — Supporting Information [file CTM2-14-e1786-s032.tif]

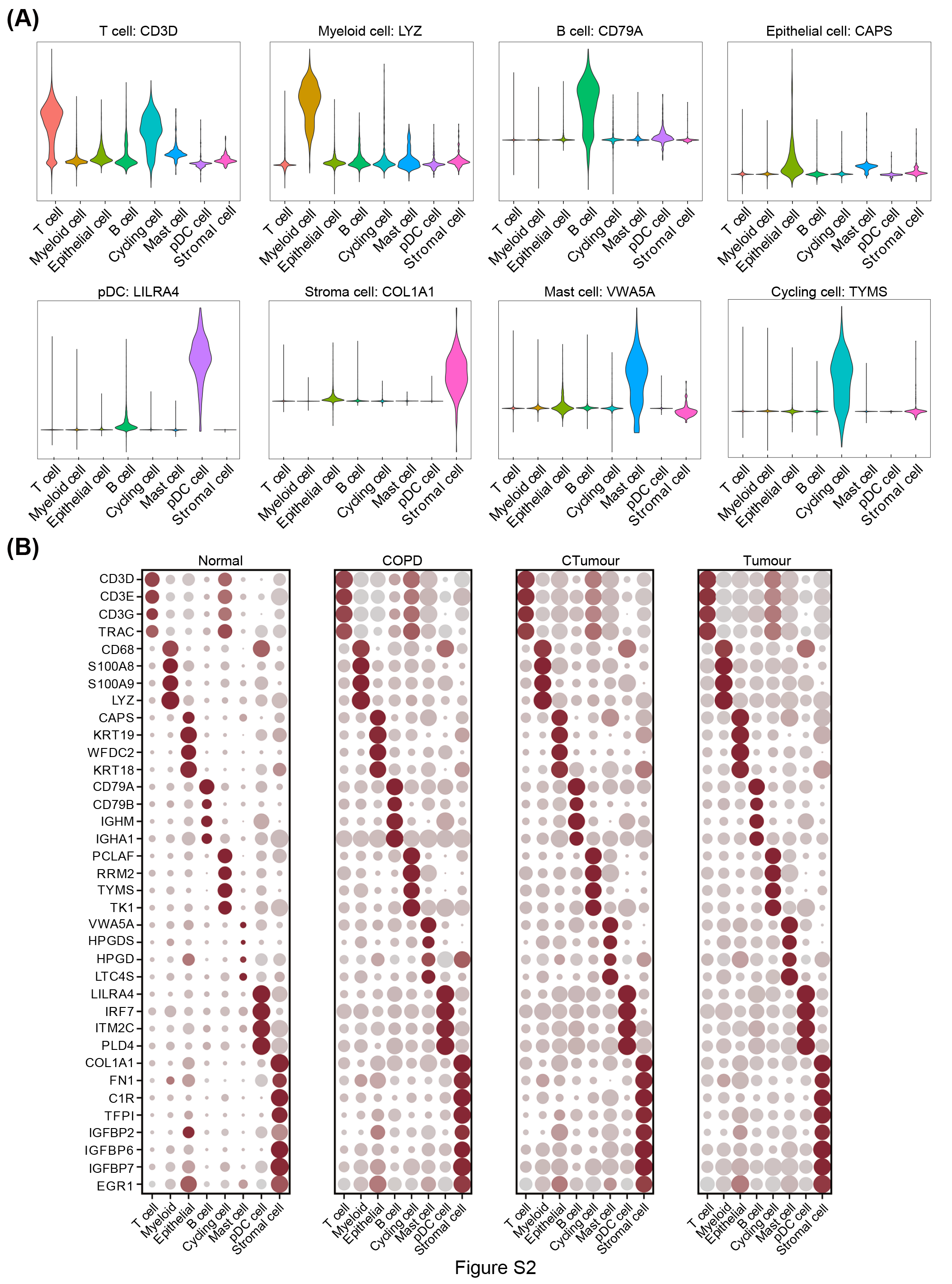

Supplement: Supplementary file 3 — Supporting Information [file CTM2-14-e1786-s023.tif]

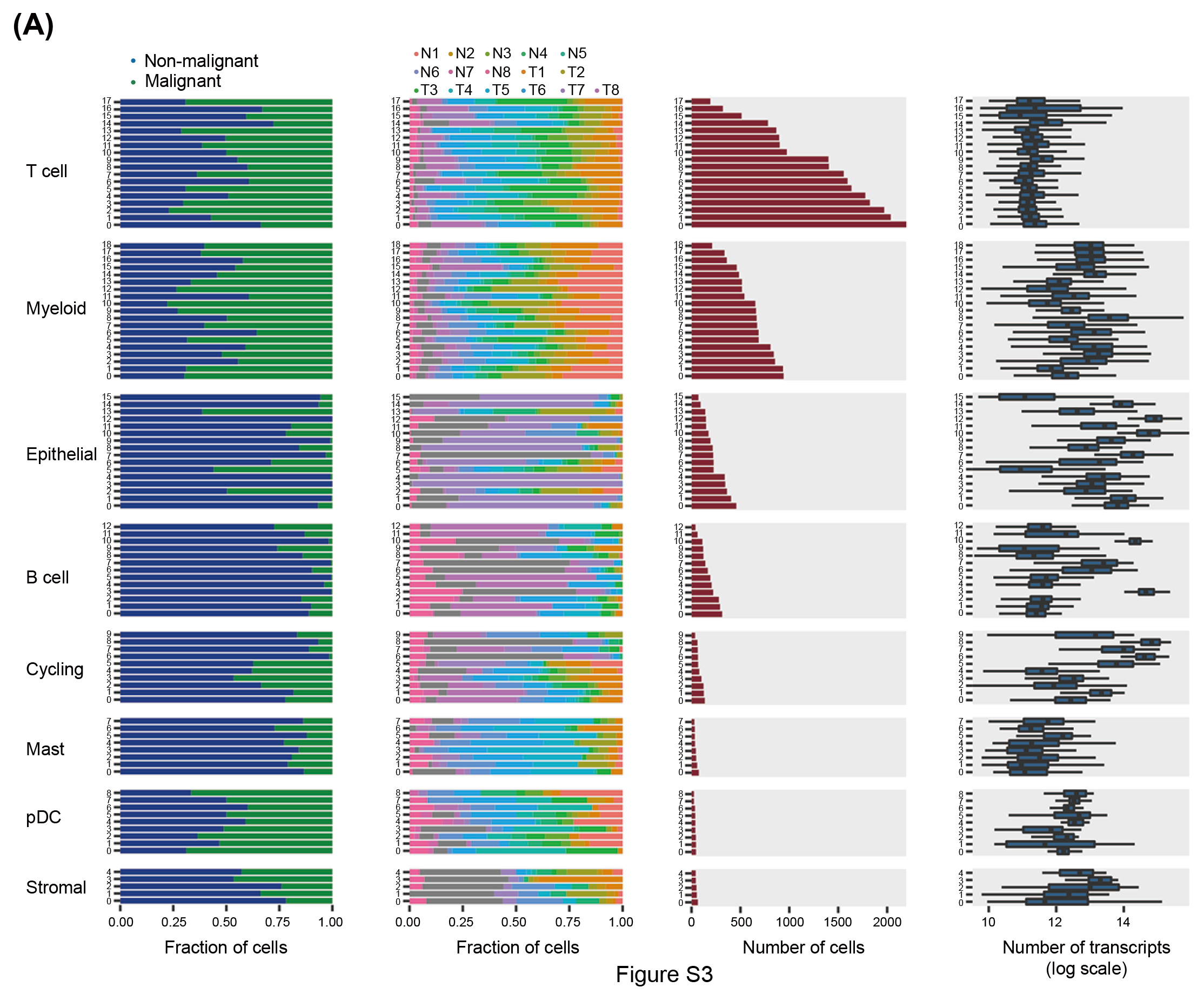

Supplement: Supplementary file 4 — Supporting Information [file CTM2-14-e1786-s019.tif]

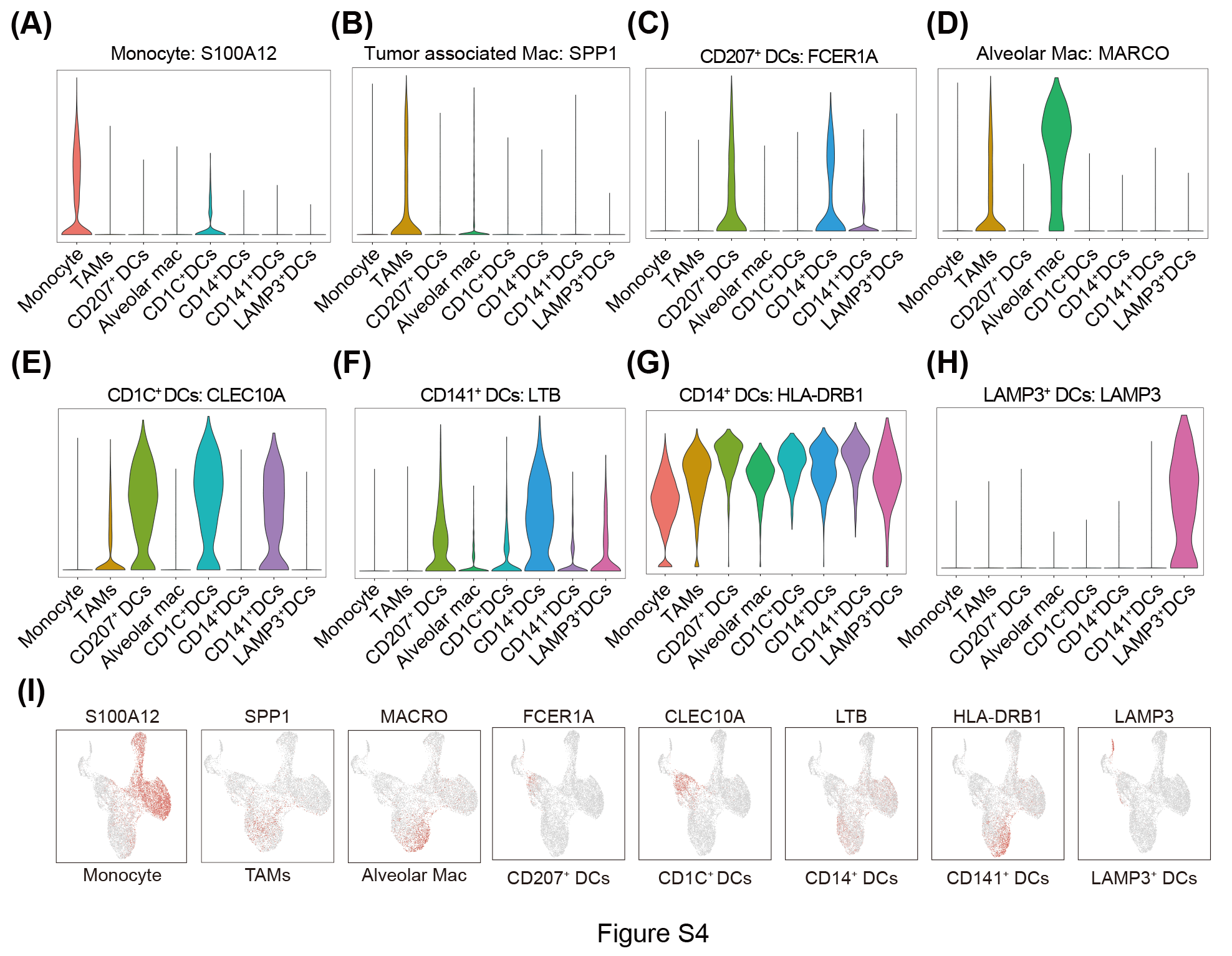

Supplement: Supplementary file 5 — Supporting Information [file CTM2-14-e1786-s021.tif]

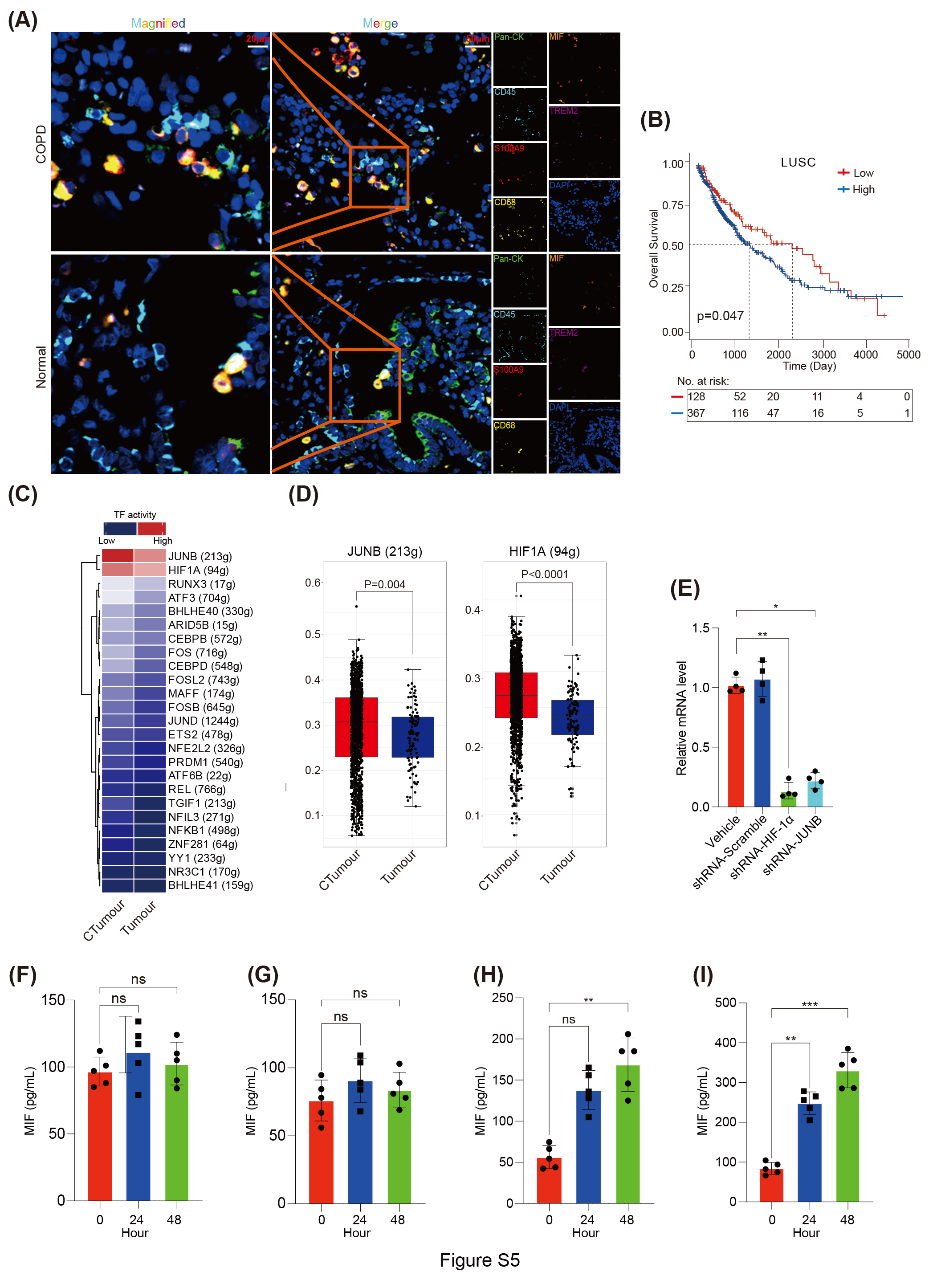

Supplement: Supplementary file 6 — Supporting Information [file CTM2-14-e1786-s028.tif]

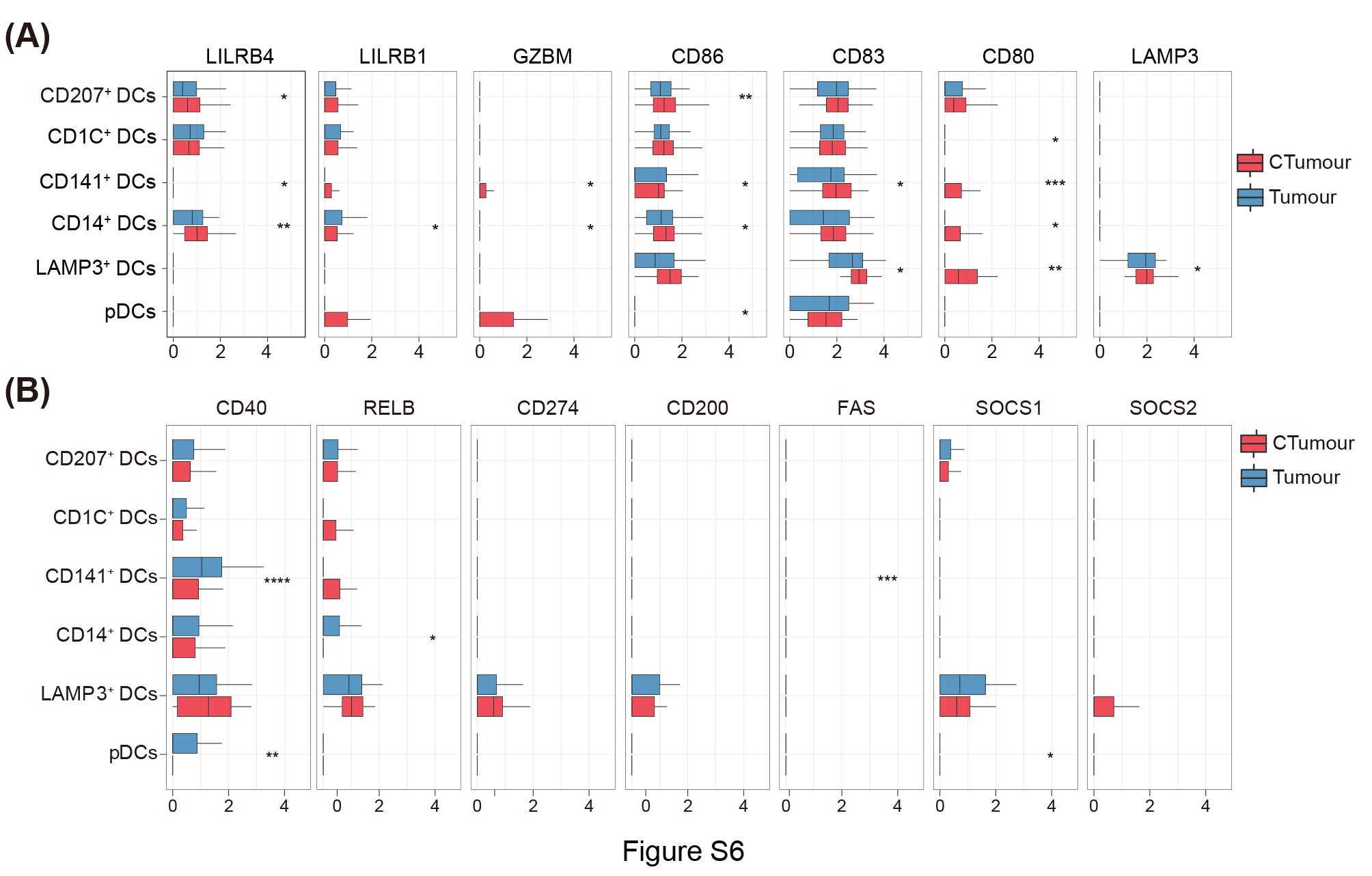

Supplement: Supplementary file 7 — Supporting Information [file CTM2-14-e1786-s002.tif]

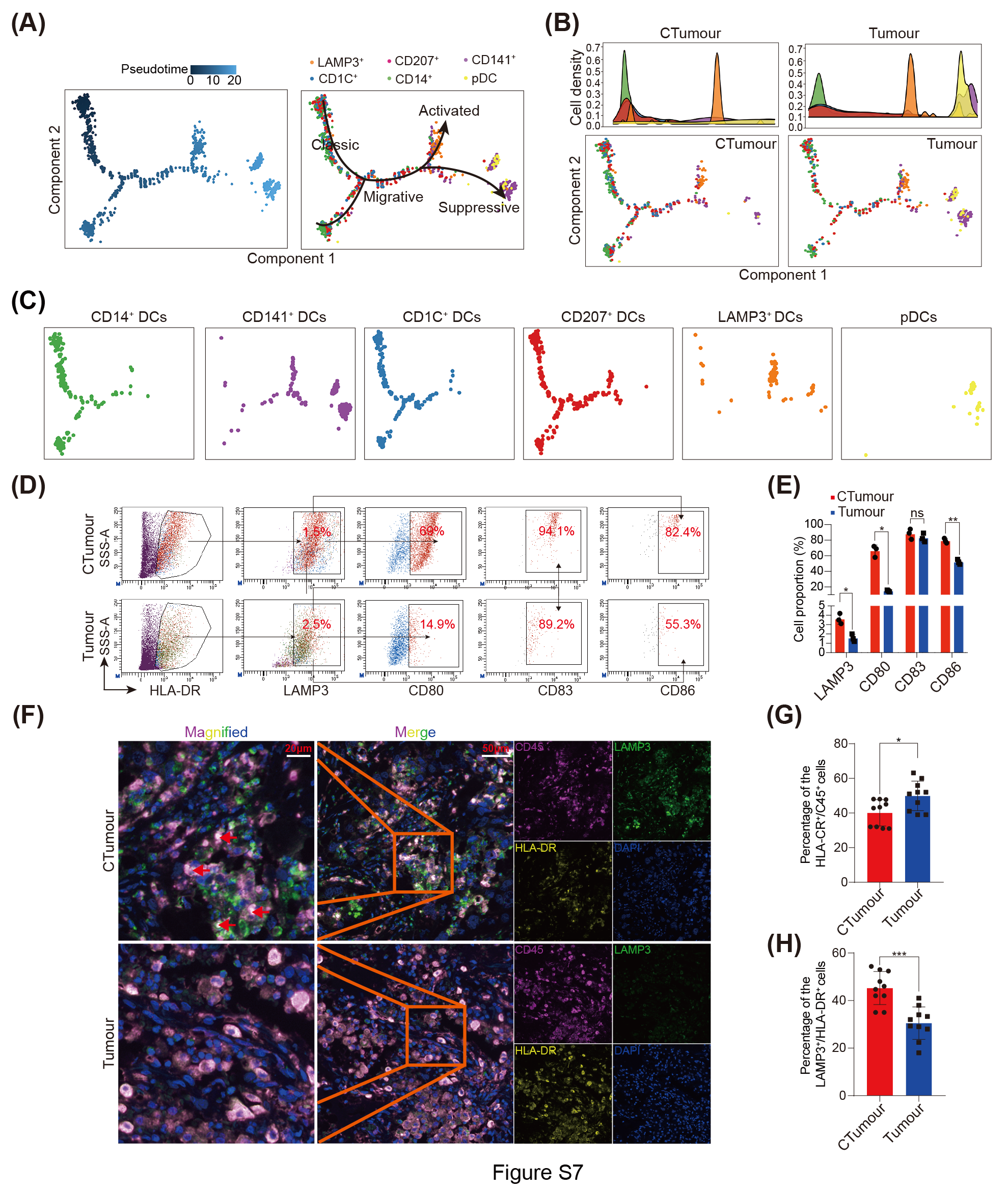

Supplement: Supplementary file 8 — Supporting Information [file CTM2-14-e1786-s006.tif]

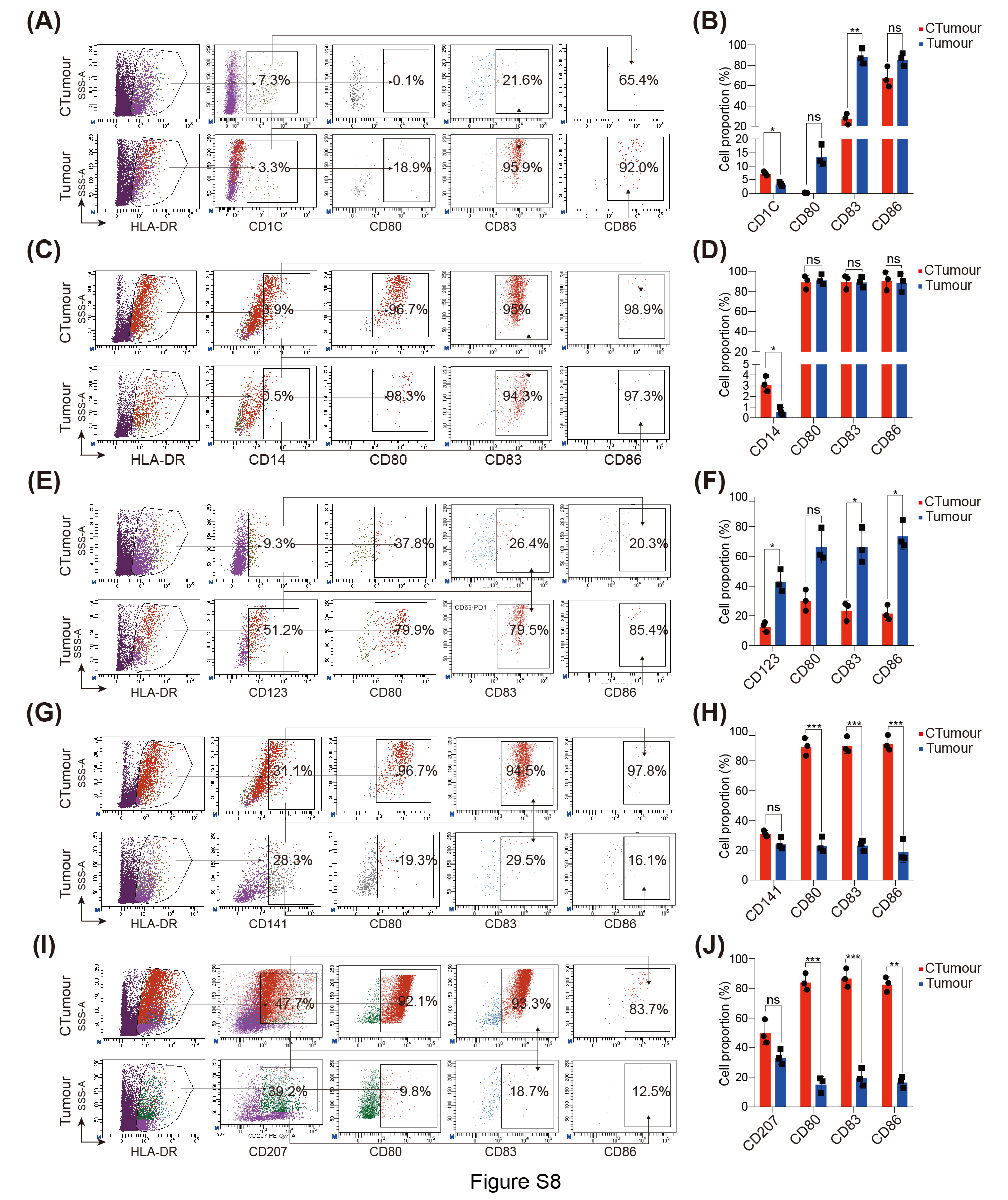

Supplement: Supplementary file 9 — Supporting Information [file CTM2-14-e1786-s009.tif]

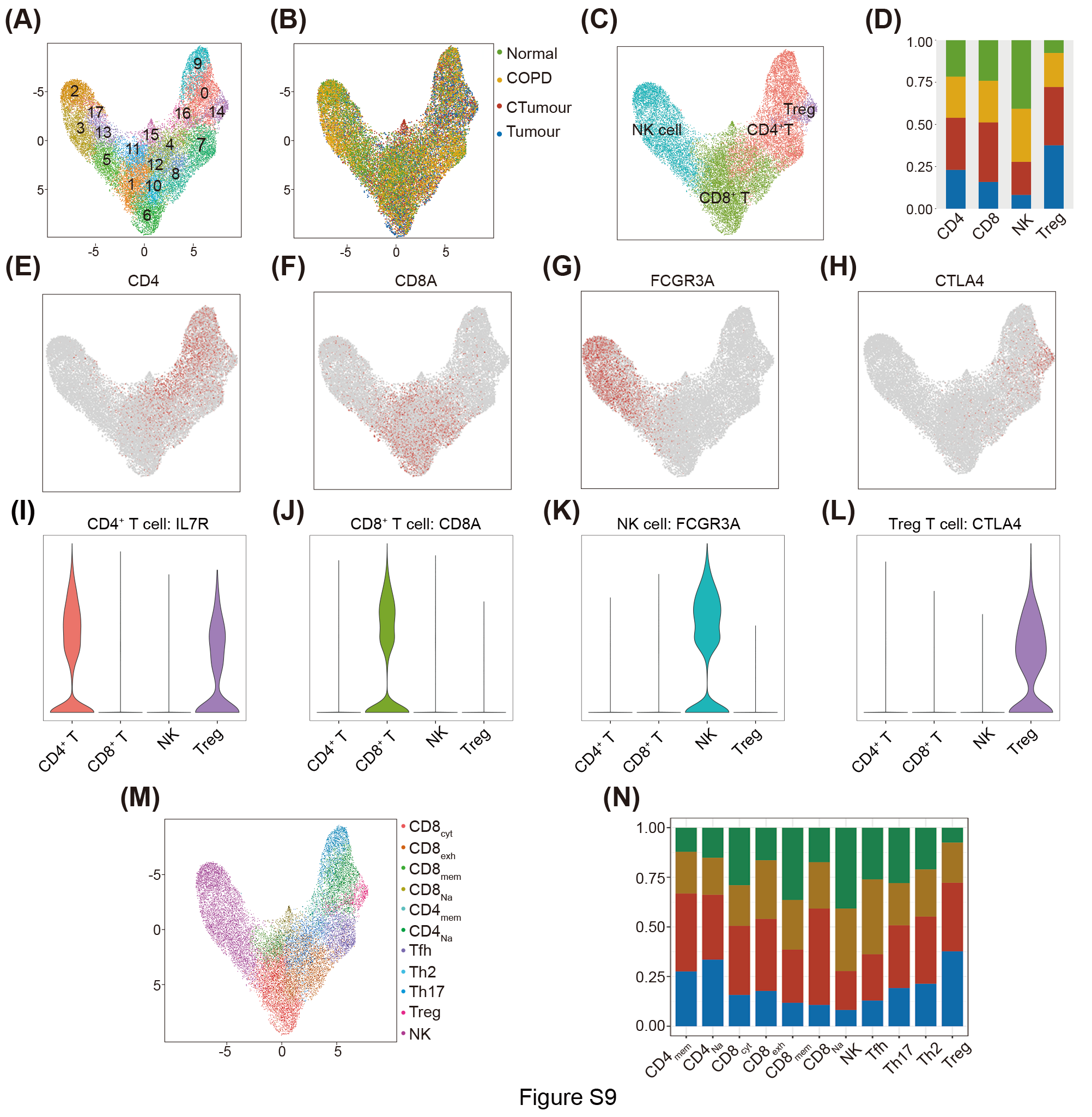

Supplement: Supplementary file 10 — Supporting Information [file CTM2-14-e1786-s013.tif]

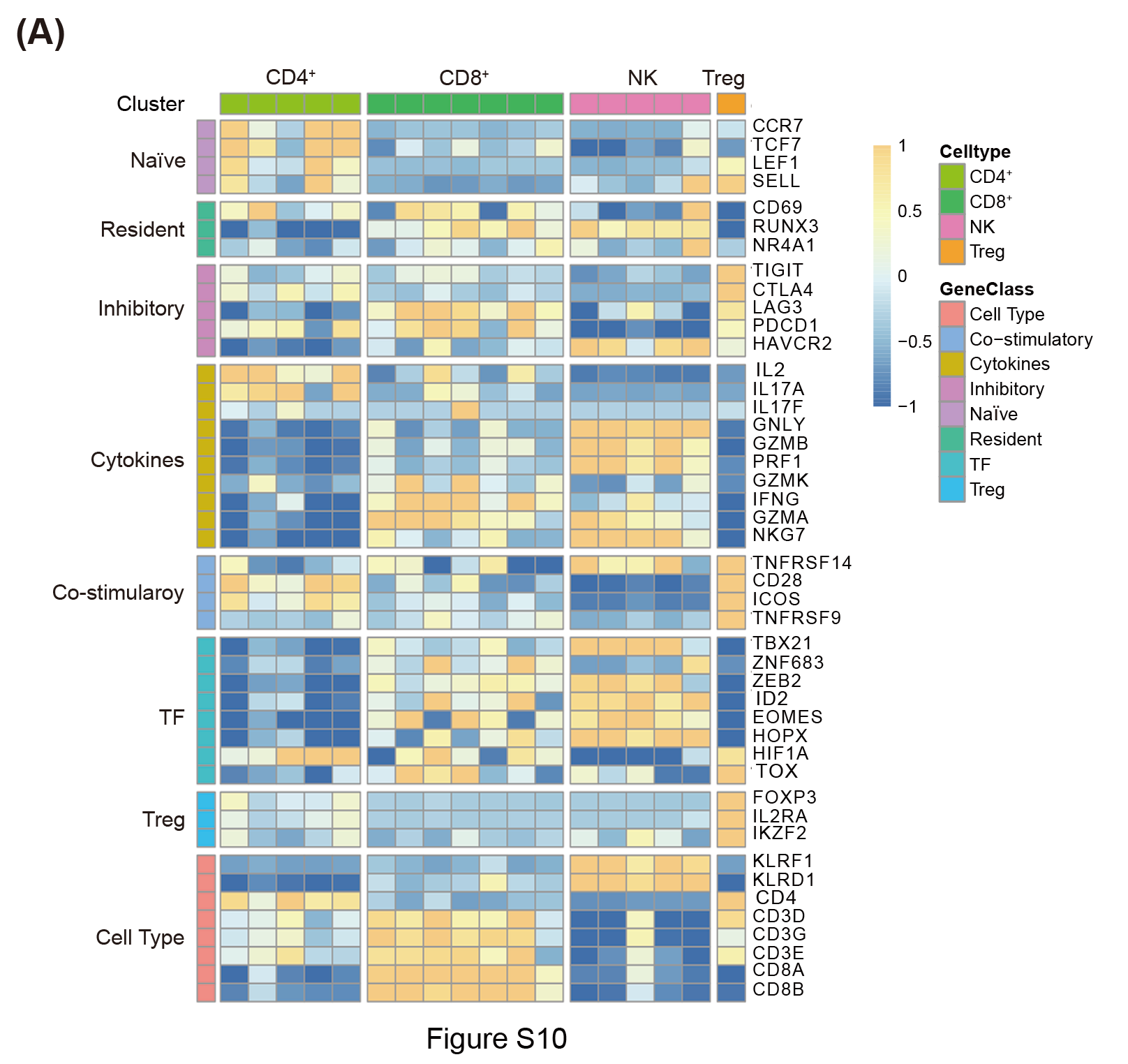

Supplement: Supplementary file 11 — Supporting Information [file CTM2-14-e1786-s029.tif]

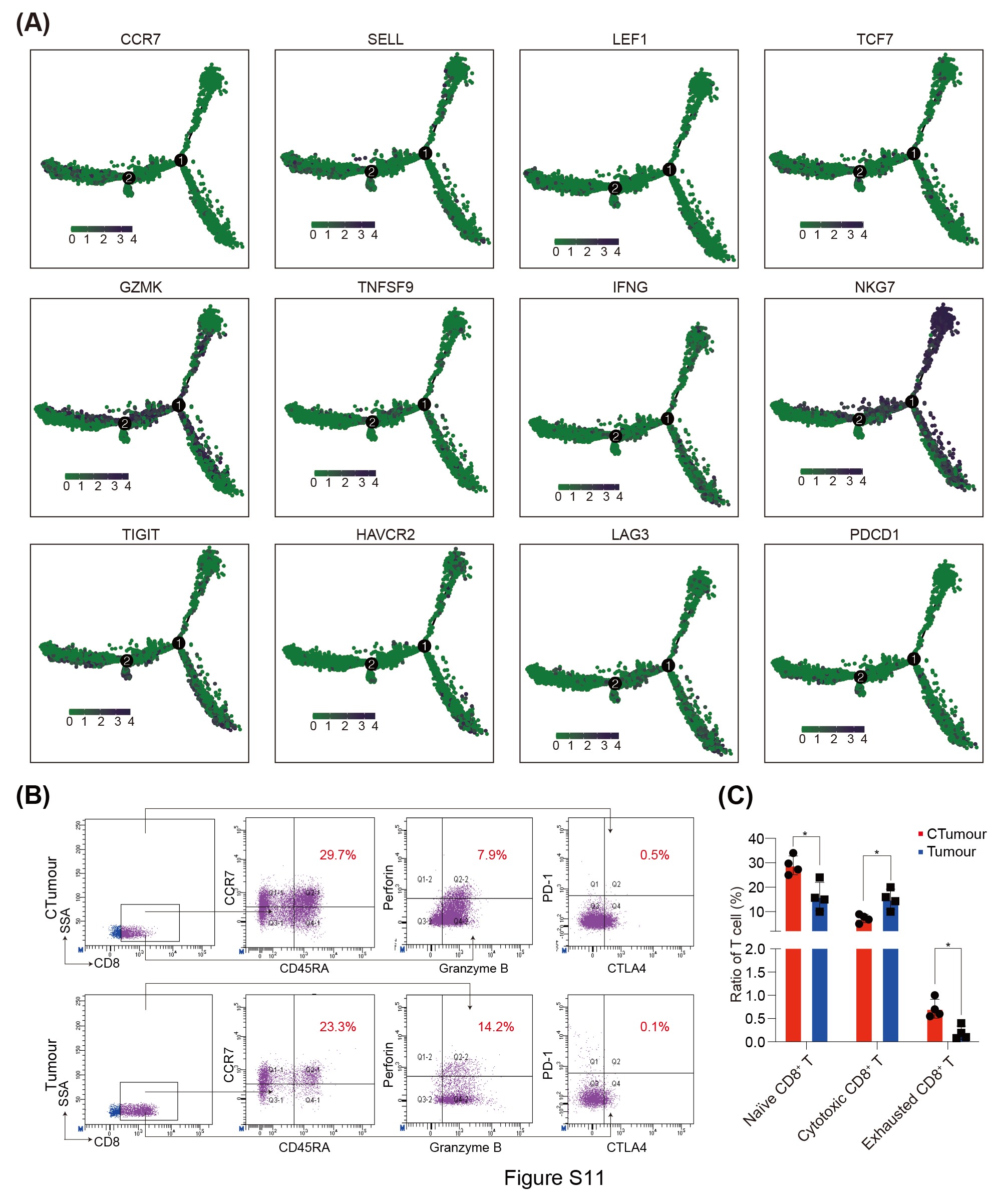

Supplement: Supplementary file 12 — Supporting Information [file CTM2-14-e1786-s024.tif]

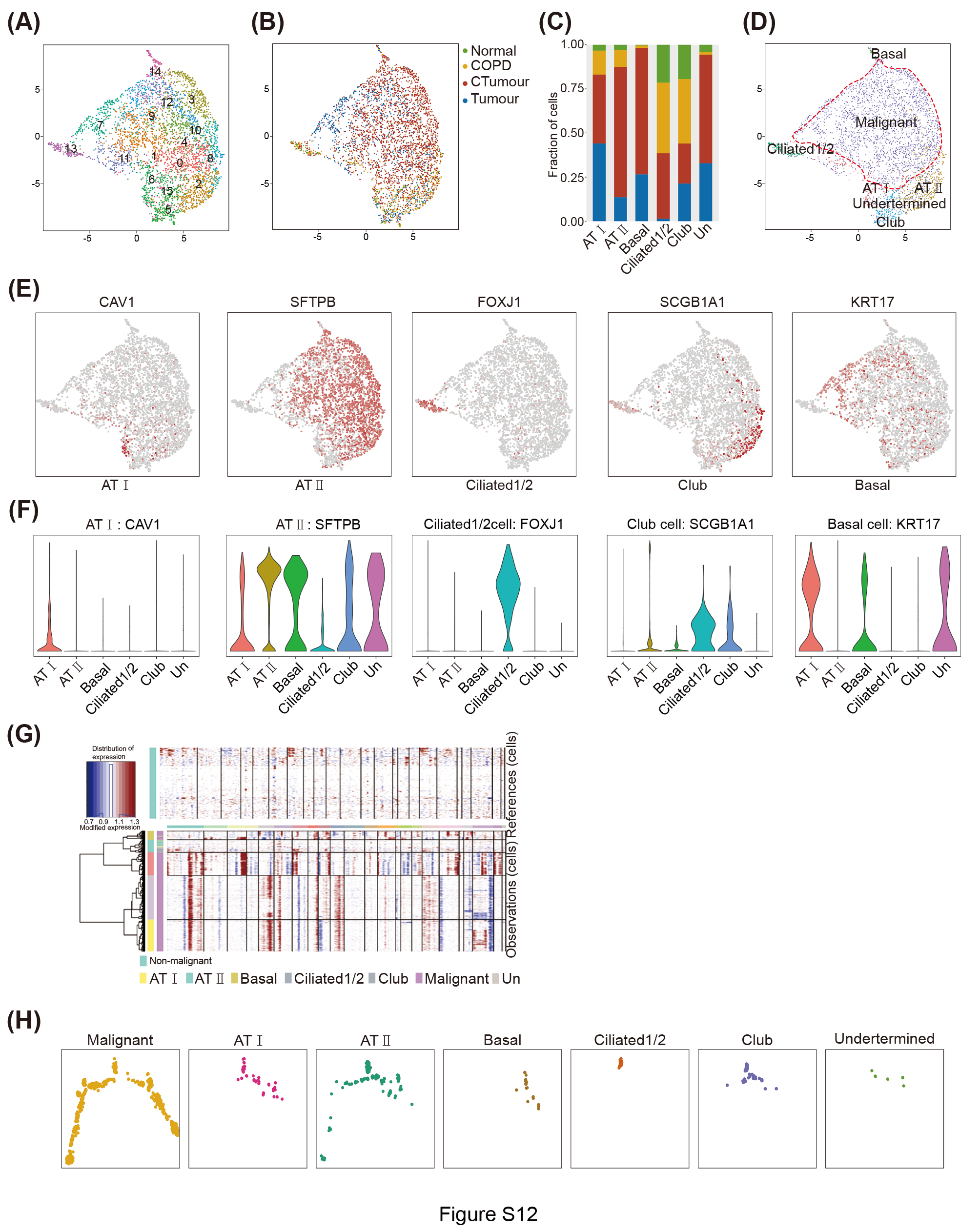

Supplement: Supplementary file 13 — Supporting Information [file CTM2-14-e1786-s001.tif]

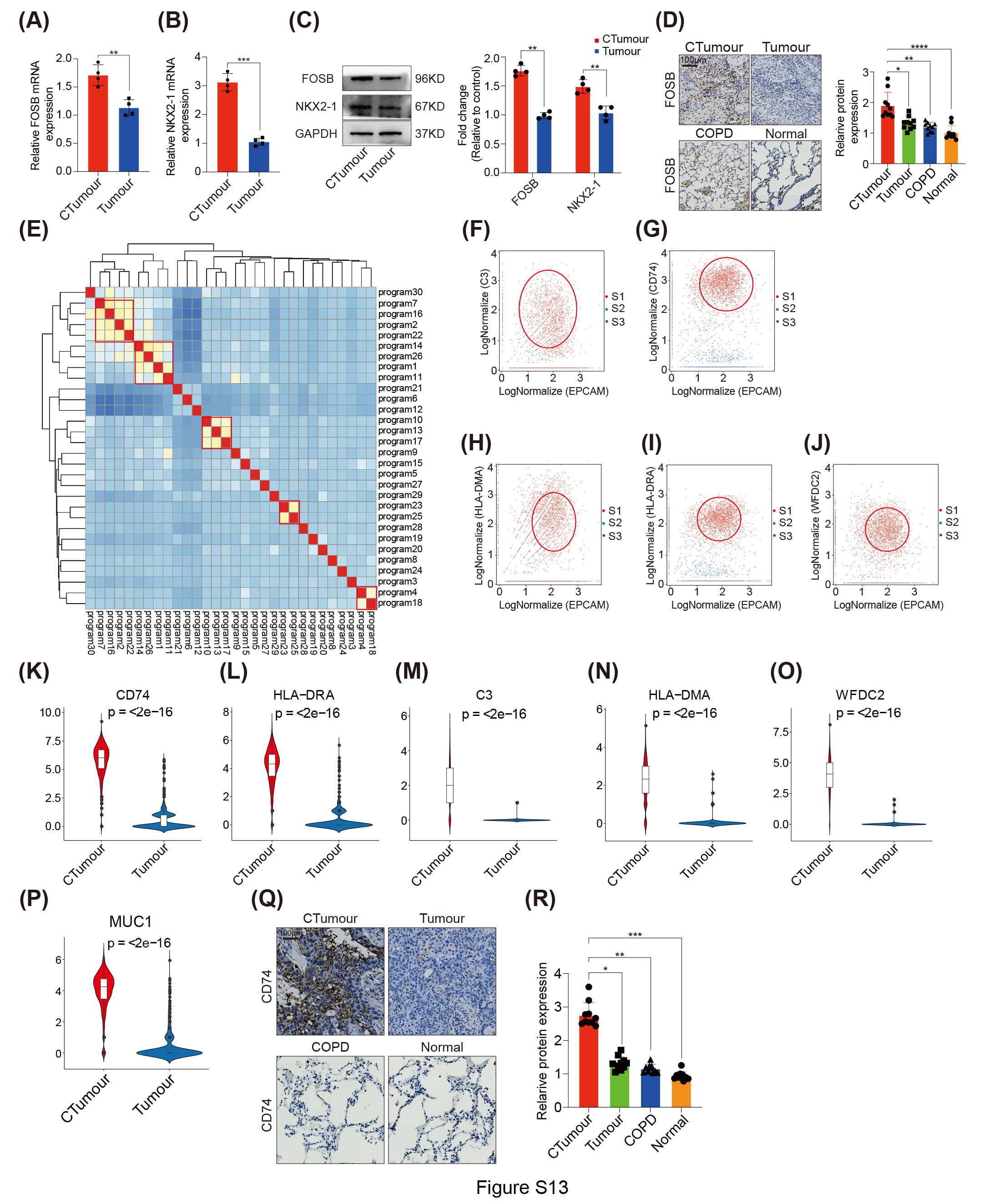

Supplement: Supplementary file 14 — Supporting Information [file CTM2-14-e1786-s018.tif]

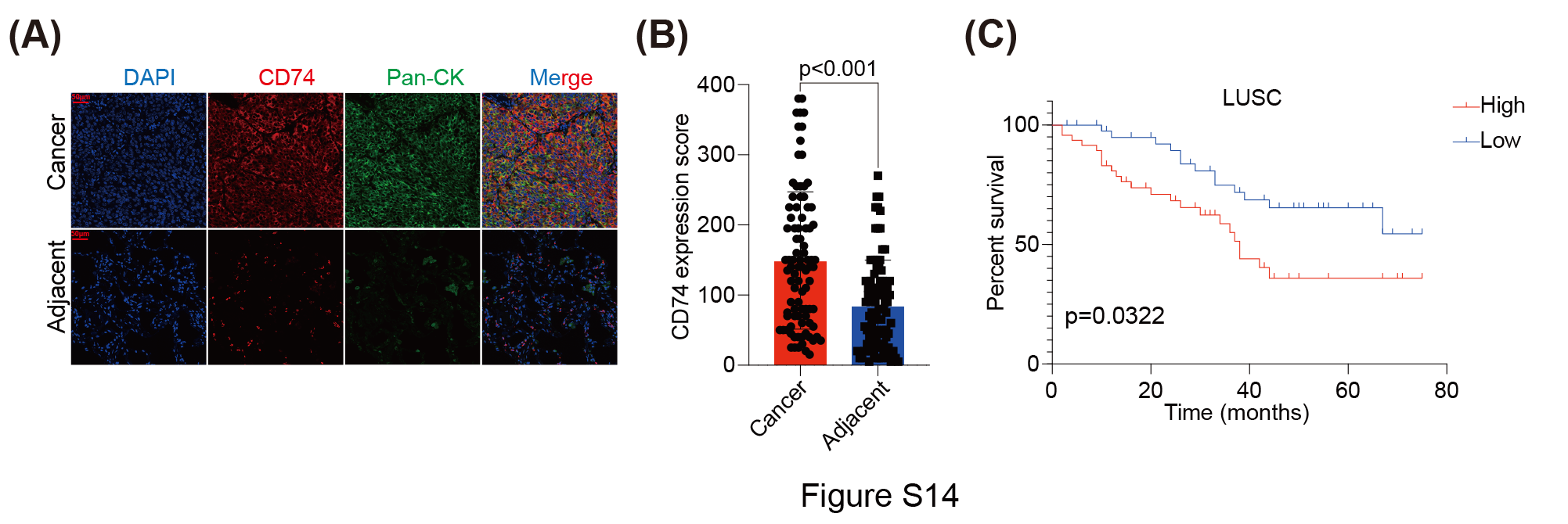

Supplement: Supplementary file 15 — Supporting Information [file CTM2-14-e1786-s015.tif]

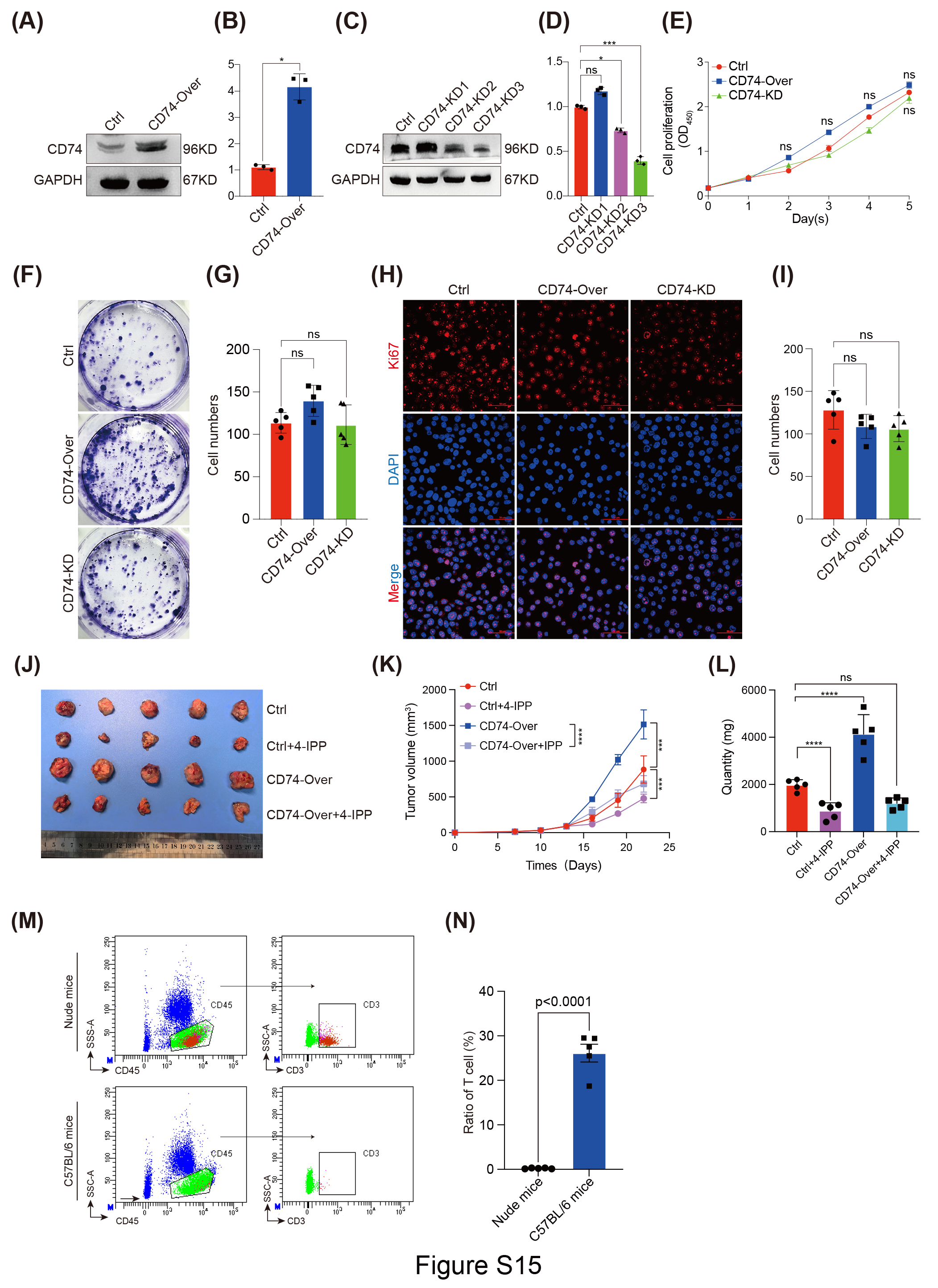

Supplement: Supplementary file 16 — Supporting Information [file CTM2-14-e1786-s020.tif]

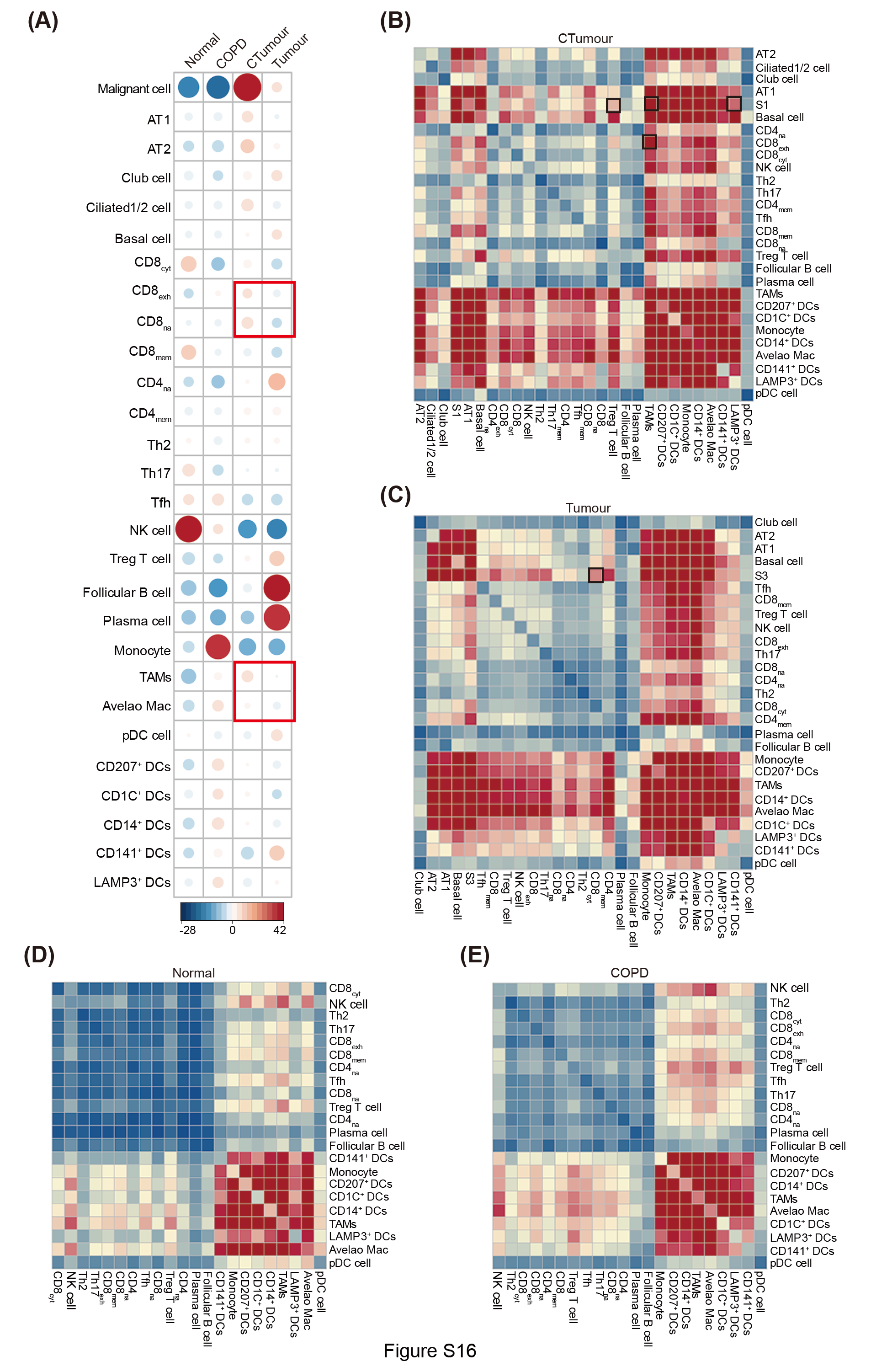

Supplement: Supplementary file 17 — Supporting Information [file CTM2-14-e1786-s008.tif]

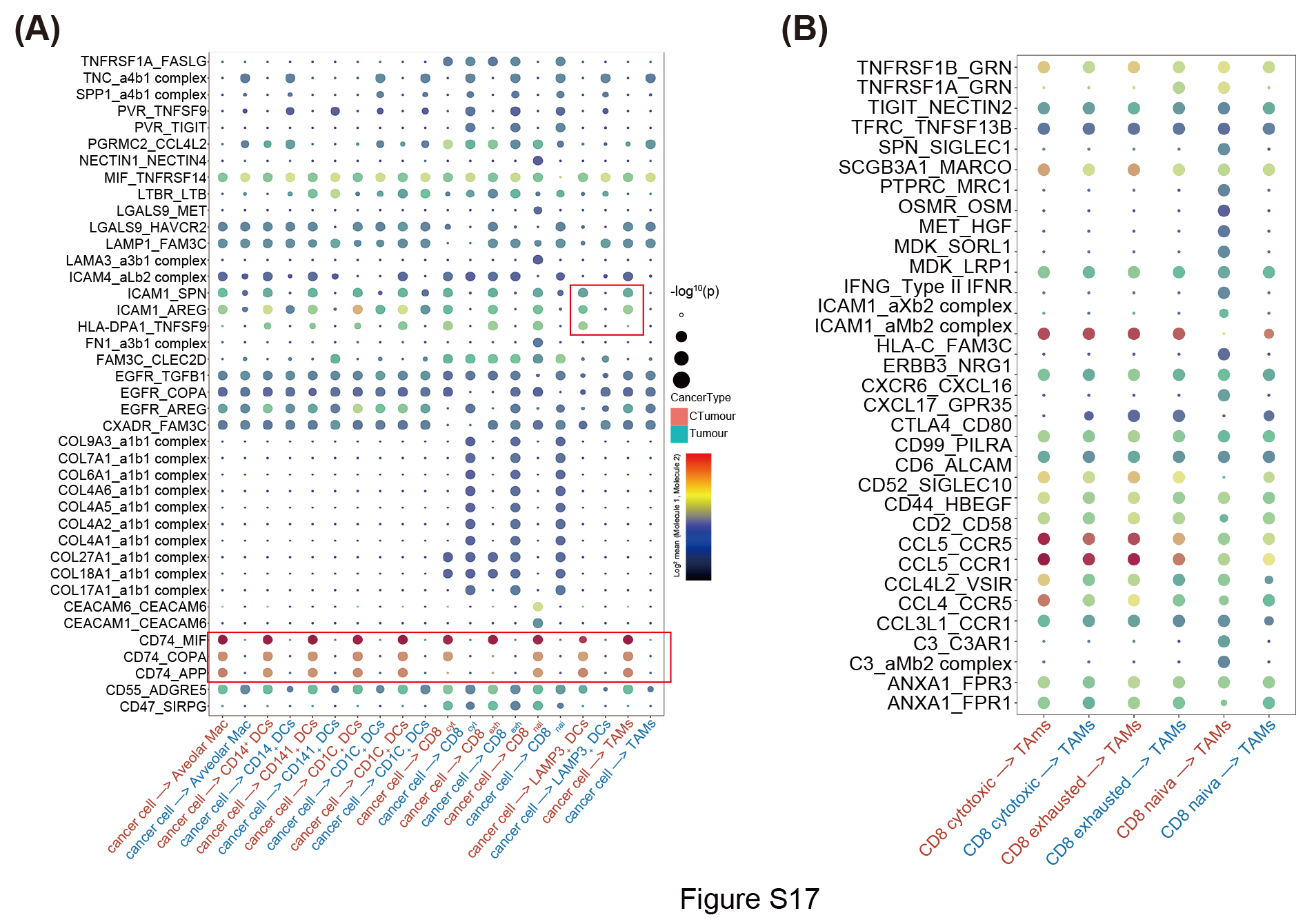

Supplement: Supplementary file 18 — Supporting Information [file CTM2-14-e1786-s005.tif]

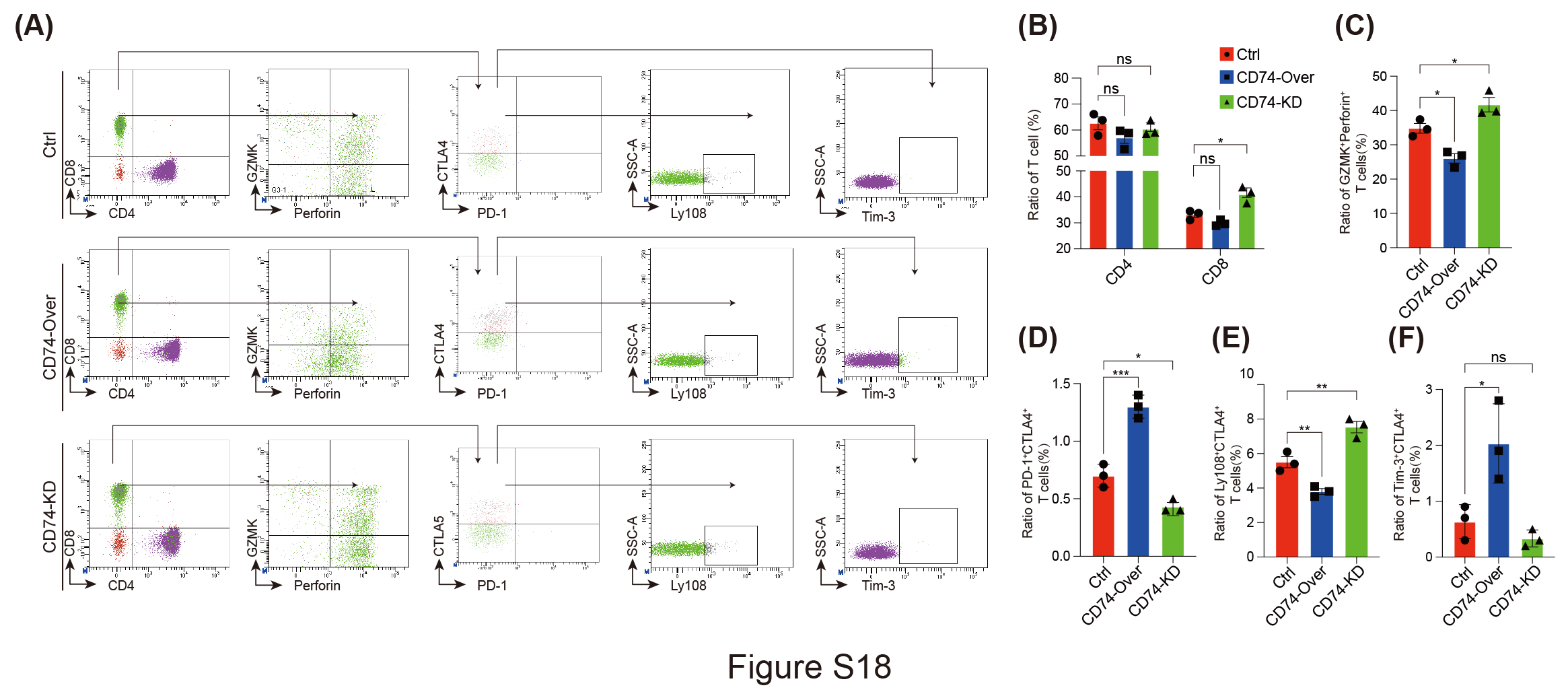

Supplement: Supplementary file 19 — Supporting Information [file CTM2-14-e1786-s031.tif]

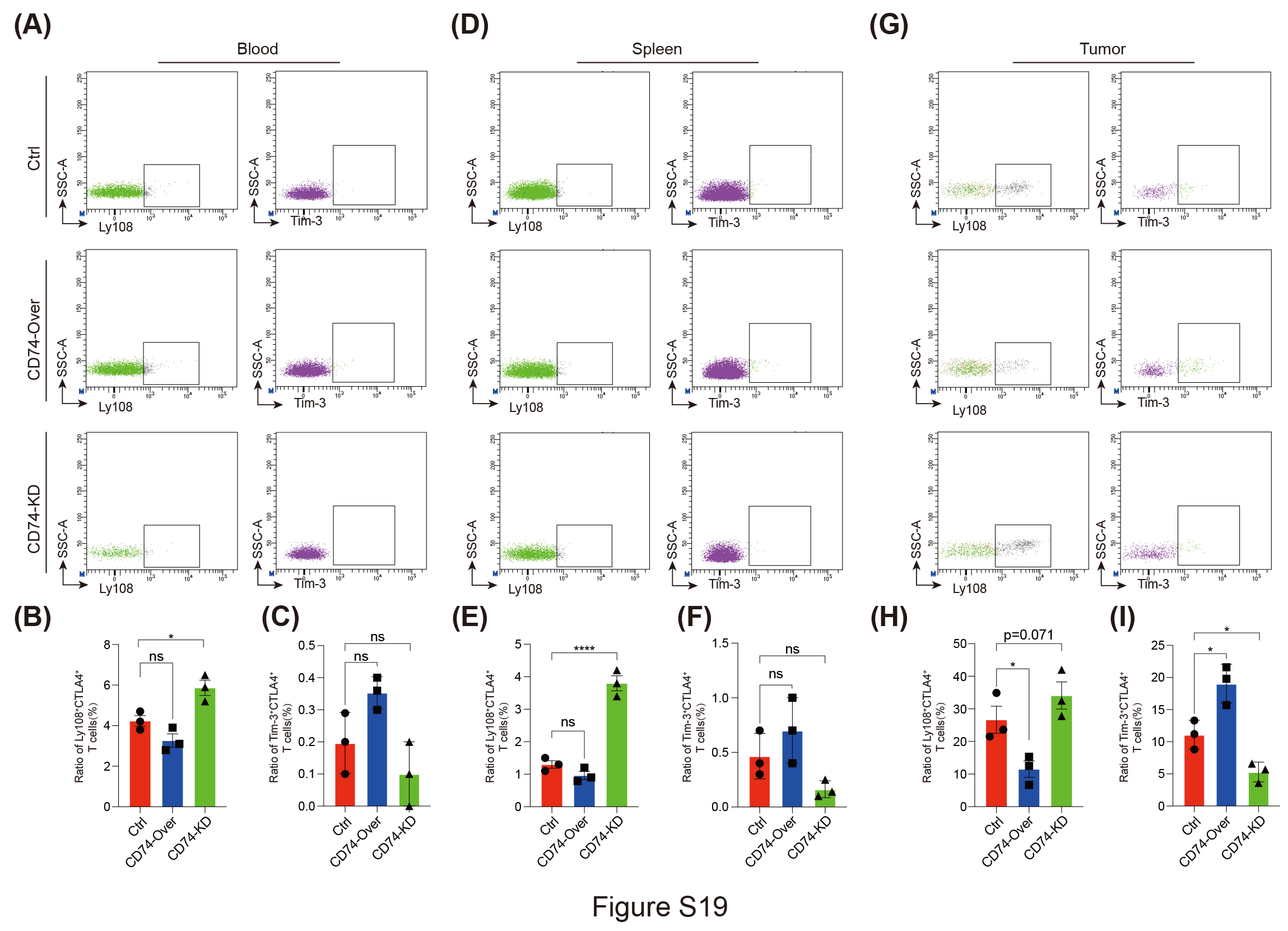

Supplement: Supplementary file 20 — Supporting Information [file CTM2-14-e1786-s010.tif]

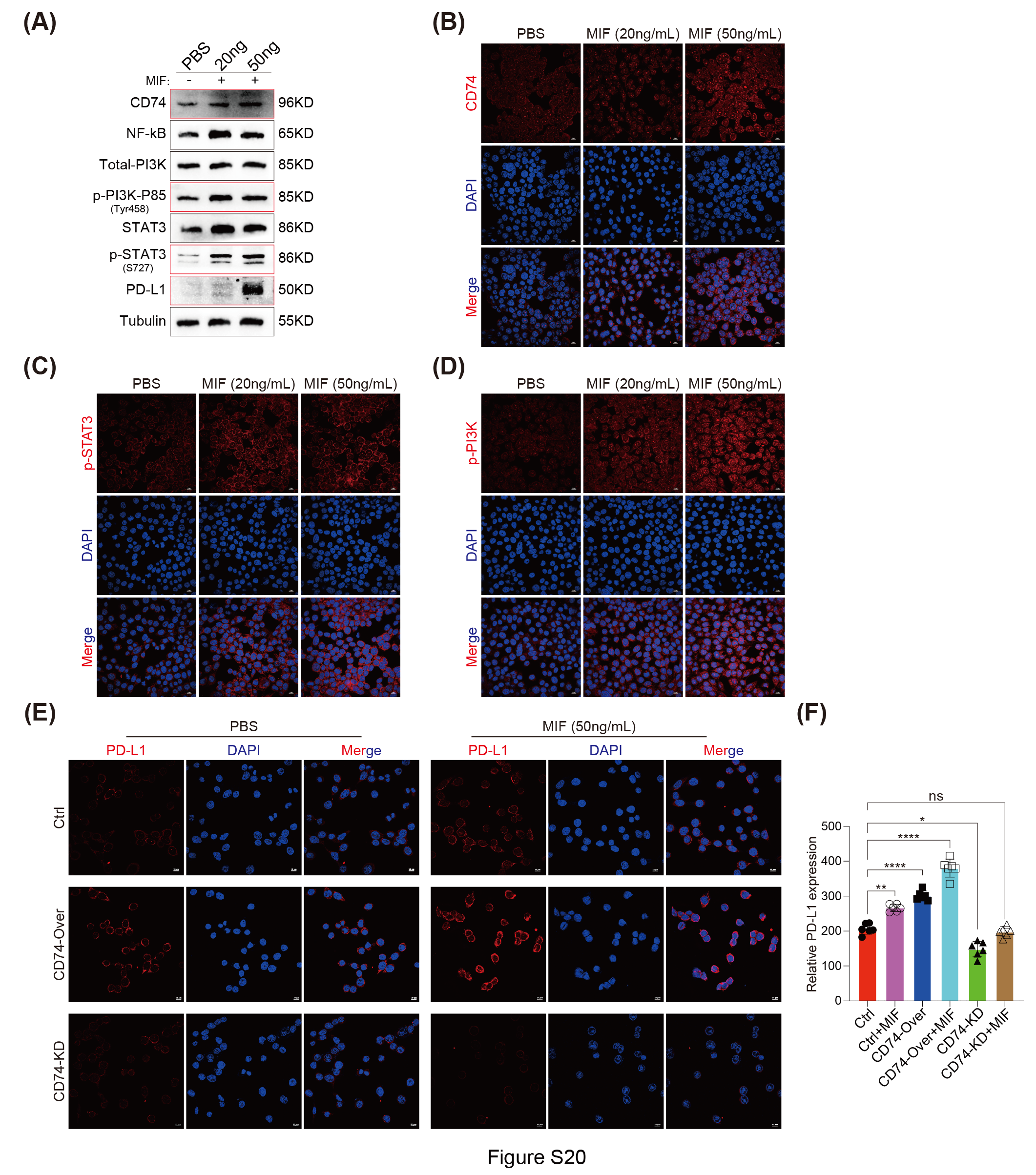

Supplement: Supplementary file 21 — Supporting Information [file CTM2-14-e1786-s007.tif]

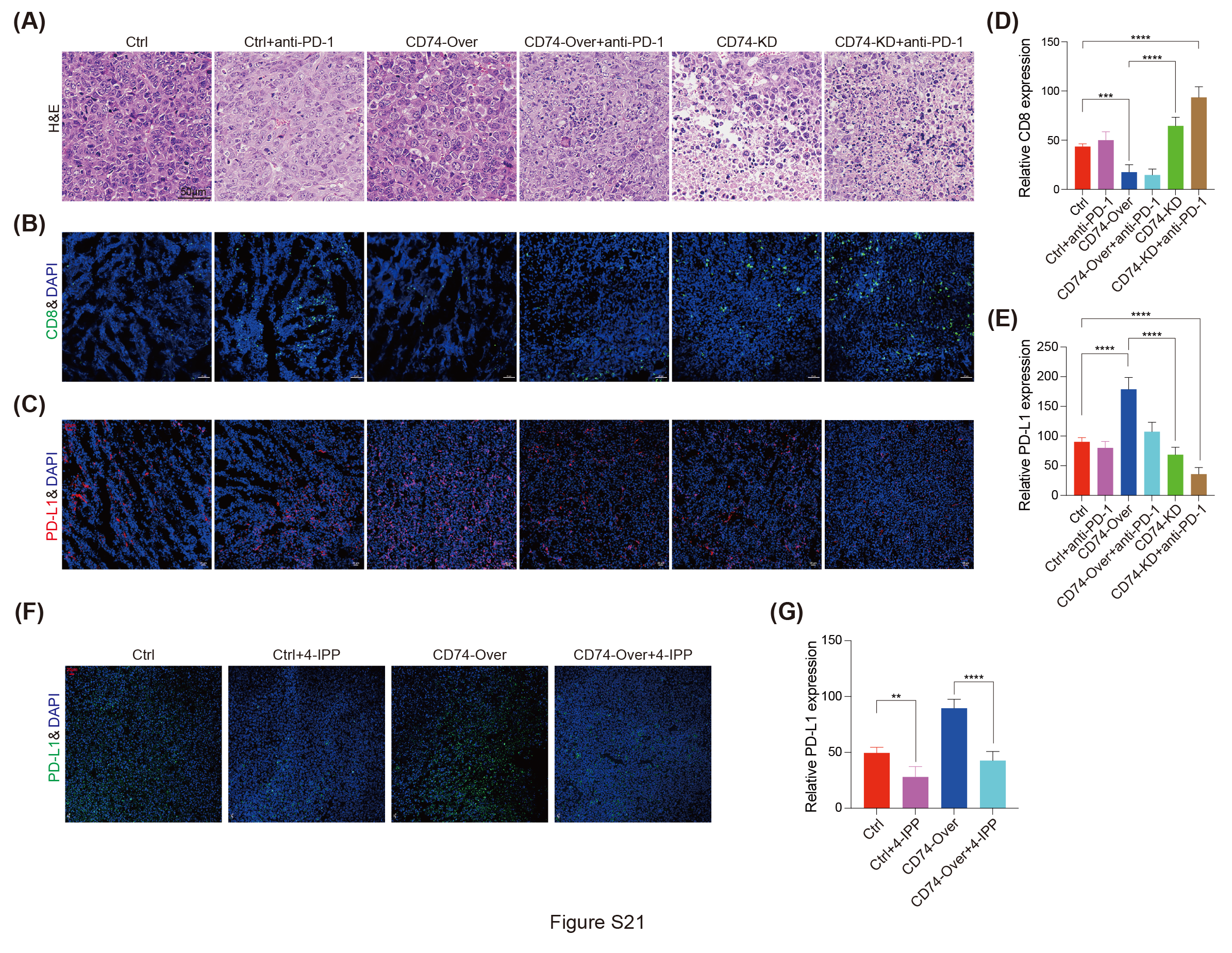

Supplement: Supplementary file 22 — Supporting Information [file CTM2-14-e1786-s014.tif]
